# Structural Covariance of Cortical Gyrification at Illness Onset in Treatment Resistance: A Longitudinal Study of First-Episode Psychoses

**DOI:** 10.1093/schbul/sbab035

**Published:** 2021-04-14

**Authors:** Olesya Ajnakina, Tushar Das, John Lally, Marta Di Forti, Carmine M Pariante, Tiago Reis Marques, Valeria Mondelli, Anthony S David, Robin M Murray, Lena Palaniyappan, Paola Dazzan

**Affiliations:** 1 Department of Biostatistics and Health Informatics, Institute of Psychiatry, Psychology & Neuroscience, King’s College London, London, UK; 2 Department of Behavioural Science and Health, Institute of Epidemiology and Health Care, University College London, London, UK; 3 Departments of Psychiatry & Medical Biophysics, Robarts Research Institute & Lawson Health Research Institute, University of Western Ontario, London, Ontario, Canada; 4 Department of Psychosis Studies, Institute of Psychiatry, Psychology & Neuroscience, King’s College London, London, UK; 5 Department of Psychiatry, Royal College of Surgeons in Ireland, Dublin, Ireland; 6 Department of Psychiatry, St Vincent’s Hospital Fairview, Dublin, Ireland; 7 Department of Psychiatry, Mater Misericordiae University Hospital, Dublin, Ireland; 8 MRC Social, Genetic & Developmental Psychiatry Centre, Institute of Psychiatry, Psychology & Neuroscience, King’s College London, London, UK; 9 Department of Psychological Medicine, Institute of Psychiatry, Psychology and Neuroscience, Kings College London, London, UK; 10 Psychiatric Imaging Group, MRC London Institute of Medical Sciences (LMS), Hammersmith Hospital, Imperial College London, London, UK; 11 Institute of Mental Health, University College London, London, UK; 12 Department of Psychiatry, Experimental Biomedicine and Clinical Neuroscience, University of Palermo, Palermo, Italy; 13 National Institute for Health Research (NIHR) Mental Health Biomedical Research Centre at South London and Maudsley NHS Foundation Trust and King’s College London, London, UK

**Keywords:** first-episode psychosis, treatment-resistant, clozapine, longitudinal, gyrification, MRI, schizophrenia

## Abstract

Treatment resistance (TR) in patients with first-episode psychosis (FEP) is a major cause of disability and functional impairment, yet mechanisms underlying this severe disorder are poorly understood. As one view is that TR has neurodevelopmental roots, we investigated whether its emergence relates to disruptions in synchronized cortical maturation quantified using gyrification-based connectomes. Seventy patients with FEP evaluated at their first presentation to psychiatric services were followed up using clinical records for 4 years; of these, 17 (24.3%) met the definition of TR and 53 (75.7%) remained non-TR at 4 years. Structural MRI images were obtained within 5 weeks from first exposure to antipsychotics. Local gyrification indices were computed for 148 contiguous cortical regions using FreeSurfer; each subject’s contribution to group-based structural covariance was quantified using a jack-knife procedure, providing a single deviation matrix for each subject. The latter was used to derive topological properties that were compared between TR and non-TR patients using a Functional Data Analysis approach. Compared to the non-TR patients, TR patients showed a significant reduction in small-worldness (Hedges’s *g* = 2.09, *P* < .001) and a reduced clustering coefficient (Hedges’s *g* = 1.07, *P* < .001) with increased length (Hedges’s *g* = −2.17, *P* < .001), indicating a disruption in the organizing principles of cortical folding. The positive symptom burden was higher in patients with more pronounced small-worldness (*r* = .41, *P* = .001) across the entire sample. The trajectory of synchronized cortical development inferred from baseline MRI-based structural covariance highlights the possibility of identifying patients at high-risk of TR prospectively, based on individualized gyrification-based connectomes.

## Introduction

Approximately 30% of patients with a diagnosis of schizophrenia spectrum disorders will experience insufficient treatment response to available treatments, a phenomenon known as treatment resistance (TR).^1,2^ In line with existing evidence,^3^ we have previously shown that around 70% of patients with first-episode psychosis, who later develop TR, already exhibit a lack of response to non-clozapine antipsychotic treatments from their first contact with mental health services, while a remaining 30% become non-responsive at later stages.^4,5^ This, in combination with evidence that a history of obstetric complications^6,7^ and a younger age of illness onset are associated with a greater risk of developing TR,^4,8,9^ suggests that neurodevelopmental disruption may play a crucial role in the pathophysiology of TR.

Alterations in cortical folding (gyrification), which is an excellent marker of the integrity of axonal connectivity during the prenatal period,^10^ as demonstrated in the classic lesional studies of Goldman-Rakic and Rakic,^11^ have been reported in patients with psychosis,^12,13^ including in association with lack of response to antipsychotic treatment.^14^ While age-related reductions in the degree of gyrification occur after birth, when compared to the effects on cortical thickness and volume, regional alterations in gyrification index with age are minimal.^15^ Regional or mass univariate whole brain analysis of morphological measures such as gyrification, reveals brain regions that are most susceptible to focal disease-related change.^16–19^ Nevertheless, in the presence of significant pathophysiological heterogeneity, as that suspected in TR, the presence of these changes may be subtle and differ across patients, leading towards the identification of null results and to inconsistency on repeated case-control sampling.^20^ Focussing on regional alterations also fails to quantify the relationship between concomitant changes across different brain areas, which is crucial to uncovering the presence of defects in synchronized maturation. Graph-based measures of structural covariance provide a more powerful mode of capturing defects in synchronized maturation.^21^ When applied to the gyrification index, this approach likely captures the state of axonal connectivity occurring during prenatal development,^22^ making it one of the most promising methods for tracing the basis of TR back to factors that predate the onset of symptoms in psychosis. Additionally, deriving network metrics for the individual patient, which is at the core of the perturbation-based network analyses that compute an individual’s distance from a group norm,^23^ further allows for quantification of the heterogeneity inherent to many clinical subgroups.

Therefore, this study investigated if the emergence of TR in the 4 years following the first episode of psychosis is already associated with alterations in individual structural covariance of cortical gyrification at illness onset. While there are multiple metrics available to study cortical structure,^24,25^ the utility of structural covariance approaches in the study of psychoses, including schizophrenia, has been established only for thickness^26^ and gyrification,^22^ and among these, gyrification appears to be less malleable to environmental influences.^22^ In this context, and in continuity with our prior work,^14,27,28^ we chose the gyrification index as the metric of interest for this study. As treatment-resistant psychosis appears to have a unique neurobiological basis, different from that of partially treatment-responsive psychosis, we used a categorical definition of TR. We hypothesized that patients with first-episode psychosis who later developed TR would already show evidence of alterations in synchronized cortical maturation at the time of their first contact with mental health services.

## Methods

### Sample Ascertainment

Participants were recruited as part of the National Institute of Health Research (NIHR) Biomedical Research Centre (BRC) Genetics and Psychosis (GAP) study conducted in South London, United Kingdom.^29^ In line with previous research,^3,30^ we included all individuals aged 16–65 years with a first episode of schizophrenia spectrum disorders [codes F20–F29], affective psychoses [F30–F33] or other psychoses as defined in the International Classification of Diseases, 10th Revision (ICD-10) manual.^31^ These diagnoses were further validated by administration of the Schedules for Clinical Assessment in Neuropsychiatry (SCAN).^32^ The study exclusion criteria were evidence of (1) psychotic symptoms precipitated by an organic cause; (2) transient psychotic symptoms resulting from acute intoxication as defined in ICD-10; (3) head injury causing clinically significant loss of consciousness; and (4) intellectual disability (IQ < 70).

### Ethics

The GAP study was granted ethical approval by the South London and Maudsley and Institute of Psychiatry Local Research Ethics Committee (reference number: 05/Q0706/158). All cases gave informed written consent after reading a detailed information sheet.

### Data at Baseline

#### Sociodemographic Characteristics.

Sociodemographic details were collated using the Medical Research Council (MRC) Sociodemographic Schedule modified version.^33^ Age at first contact with services was defined as age at which a patient was first in contact with mental health services due to their psychotic symptoms.^34^ Ethnicity was self-ascribed using the 16 categories of the UK Census in 2001 (www.statistics.gov.uk/census 2001).

#### Clinical Assessments.

Duration of untreated psychosis (DUP) was defined (in weeks) as the difference between the date of onset of psychotic symptoms and the date of treatment with antipsychotic medications.^35,36^ The Global Assessment of Functioning (GAF) scale was used to measure both overall symptoms severity and functional disability at study entry.^37^ The GAF was completed from face-to-face interviews with good inter-rater reliability (*κ* = 0.90). The degree of psychopathology over the preceding week was evaluated with the 30-item Positive and Negative Syndrome Scale (PANSS),^38,39^ in face-to-face interviews. Each PANSS item was rated on a 7-point scale (1 = absent, 7 = extreme), across all 3 subscales: positive symptoms (7 items), negative symptoms (7 items), and general psychopathology (16 items).

### MR Image Acquisition and Processing

To ensure minimal exposure to antipsychotic medications in patients, MRI scans were obtained within a 3-month period following the first contact with psychiatric services. A 3 Tesla GE (General Electric, Milwaukee) Signa HDx scanner at the Centre for Neuroimaging Sciences (CNS), Institute of Psychiatry, Psychology and Neuroscience (London, UK) was used to acquire 3-dimensional MPRAGE volumetric scans (matrix size of 256 × 256 × 166 voxels, with in-plane voxel size of 1.02 × 1.02 mm and a slice thickness of 1.2 mm (echo time/repetition time/inversion time = 2.848/6.988/650 ms, excitation flip angle 20°, one data average). Full brain and skull coverage was required for the MRI datasets and detailed quality control was carried out on all MR images according to previously published criteria.^14,27,40^ A summary of the quality control criteria used is presented in the supplementary material.

#### Cortical Gyrification Analysis.

**Fig. 1.**
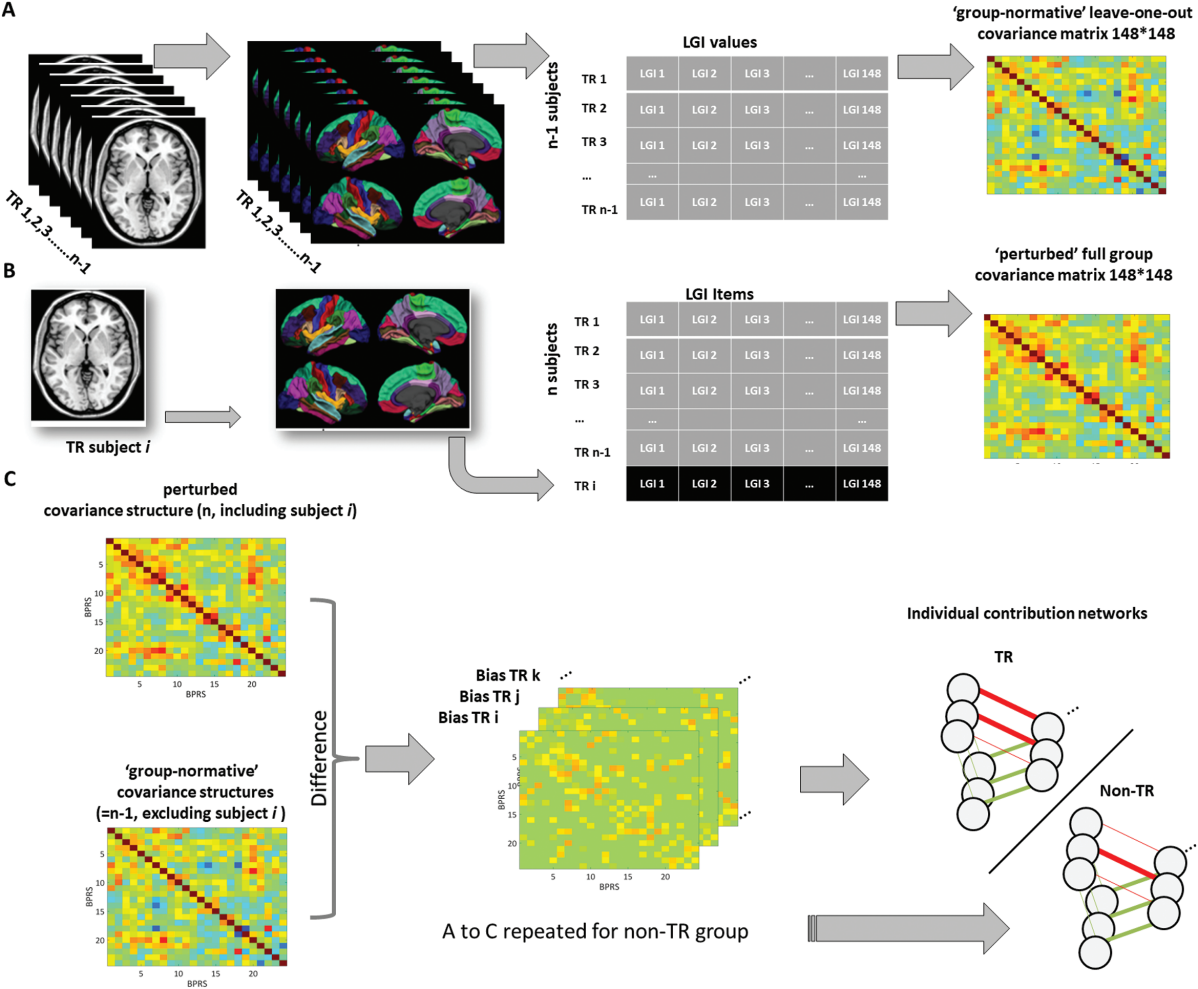
Depicts 3 steps (referred to as A, B, and C) followed to construct an individual-specific LGI network in the present study. (A) For a single group of (N-1) subjects (treatment resistance [TR] or non-TR), a specific group-based network is constructed by the correlations between LGI values based on regional LGI measures from 148 parcellations in this group, with the exclusion of a single subject i. This group network (based on N-1 matrix) has the “normative” covariance structure of that group’s gyrification pattern. (B) A new subject i belonging to that patient group is added to the group, and the perturbed network with this additional individual is constructed in the same way as the (N-1) matrix. The difference between the (N) and the (N-1) network is due to the individual i (or j or k…). (C) An individual contribution-based network is constructed using the difference of the corresponding edge between the (N) and (N-1) matrix. For illustrative purposes, only one group (TR) and only 3 nodes are shown. LGI-based networks in this study were made of 148 nodes, each node representing a single region of the parcellation scheme.

We computed local gyrification indexes (LGIs) for various anatomically defined sulcal and gyral regions of the cortical mantle using Freesurfer (FreeSurfer, version 4.5.0; http://surfer.nmr.mgh.harvard.edu/), based on the method by Schaer et al.^41^ The steps for constructing an individual-specific LGI network are presented in figure 1; more details on surface extraction are provided in the supplementary material. LGIs were computed for 148 parcellated brain regions (74 in each hemisphere; no subcortical structures included) according to the atlas of Destrieux et al^42^ and in line with our prior work on structural covariance.^14,27^ All pre-processing steps followed the standard description given by Fischl et al.^43^ This included skull-stripping, intensity correction, determination of the gray-white matter boundary followed by tessellation to generate multiple vertices across the whole brain and the expansion of this boundary to re-create the pial surface, followed by spherical morphing and registration using sulcogyral landmarks. Schaer’s automated vertex-wise method computes Zilles’ gyrification index,^44^ a ratio of the inner folded contour to the outer perimeter of the cortex, using a 25 mm hull surface around each vertex of the reconstructed pial surface.^41^ Averaging the gyrification values assigned to the vertices within each anatomical boundary of Destrieux parcellations provided the regional LGI measure for each of the 148 parcellated brain regions. An LGI value closer to 1 suggests that the region has an approximately flat pial surface with almost no “buried cortex” in sulcal ridges.

#### Constructing Gyrification-Based Individual Covariance Networks.

We first generated a 148 × 148 correlation matrix based on LGI values, separately for the 2 study groups. We estimated the contribution of each individual to their group matrix using a jack-knife bias estimation procedure, in line with our prior study.^45^ Bias values for each cell in the matrix of an individual subject quantified the contribution of that subject to the structural covariance of their group. Higher values signify greater relationship between the 2 given nodes in that subject, relative to the group norm. As a result, everyone’s matrix is a representation of the absolute contribution of that individual to their group’s overall covariance structure. This approach is mathematically similar to individual-specific gene expression networks used in bioinformatics^23,46^ and cancer studies.^47^ The bias values that constitute the edges in a group perturbation-based deviance matrix show a smooth, symmetric distribution around the absolute group correlation value, enabling parametric inferences, as shown by Liu et al.^46^ We then used the Graph Analysis Toolbox,^48^ to generate binary undirected graphs with a range of network thresholds based on connection density (ie, 0.05–0.25, with interval steps of 0.01). The interval steps were chosen to generate sufficient data points (20) for the functional data analysis (FDA) described below. This proportional thresholding and binarization based on edge density ensured that differences in absolute bias values between the 2 groups (that relate to the degree of freedom in jack-knife estimates) did not influence the estimates of topological parameters. The proportional thresholding range was chosen to enable between-group comparisons without inducing disconnection (percolation threshold) or losing small-worldness (randomness threshold) in any of the groups.^49^ Topological properties become unstable when sparsity levels are too high as some nodes are fully disconnected, while weak pairwise relationships that are likely to introduce randomness are included at lower sparsity values.^49^ See figure 1 for further details.

### Tracing Patients at Follow-up

Approximately 4 years (mean = 3.8, SD = 1.4; range = 1–9, 283 person years) after the first contact with psychiatric services, we sought to trace all patients who had given consent for their clinical records to be accessed for research purposes. Follow-up data on illness course were extracted retrospectively from electronic clinical records, the primary clinical record keeping system within the Trust,^50^ using the WHO Life Chart Schedule (LCS) extended version,^51,52^ with has good reliability.^53^ All deaths and migrations up to, and including those that occurred during, the final year of follow-up were identified by a case-tracing procedure with the Office for National Statistics (ONS) for England and Wales and the General Register Office (GRO) for Scotland.

#### Definitions of TR.

Patients were defined as having TR if they had been treated with clozapine and/or they showed little or no symptomatic improvement to 2 consecutive treatments with antipsychotic medications of adequate dose and duration (at least 6 weeks) during the follow-up period.^4,54^ The presence of no symptomatic improvement following antipsychotic treatment was established if (1) having been treated with an antipsychotic medication of adequate dose and for an adequate duration, patients did not show improvement in their clinical presentation as recorded by treating clinicians, and/or (2) the documented reason for switching antipsychotic medication was a lack of therapeutic response, and not intolerance to antipsychotic medications or self-discontinuation of medications.^4^ An adequate daily dose of antipsychotic medication was defined as a daily dose of at least 400mg of chlorpromazine equivalents.^55^

### Data Analysis

#### Confirmatory Factor Analysis.

As the Wallwork/Fortgang’s model^56^ offered the most robust model for exploring symptom profiles, we conducted a confirmatory factor analysis (CFA) to identify and evaluate the statistical fit^57^ of these symptom dimensions in our sample. The Goodness-of-Fit Index (GFI) statistics included the comparative fit index (CFI; values greater than 0.90 indicate good model fit), the root mean square error of approximation (RMSEA; values less than 0.06 indicate good model fit), and the standardized root mean square residual (SRMR; values less than 0.08 indicate good model fit).^57^ To improve the model fit, we further included correlated measurement errors into the model, based on significantly correlated residuals as indicated by modification indices.^58^ With this approach, 5 core symptom dimensions (positive, negative, excited, disorganized/concrete, and depressed^59^) were estimated for each patient.

#### Topological Analyses.

Global network properties were quantified using small-worldness (σ), characteristic path length (λ) and clustering coefficient (ϒ). While there are multiple measures of network properties available, we chose a minimal set with an index reflecting the “segregation” that may result from modular (communal) development of gyrification within a cluster of regions that increases clustering (ϒ); and an index of “integration” indicating a coordinated maturational process in gyrification across the entire brain, that reduces path length (λ). The presence of high segregation in the context of optimum integration gives rise to small-worldness, measured using the small-world index (σ). Network measures were normalized to equivalent values derived from 20 random (“null”) networks with the same degree of distribution, in line with previous studies.^45,60^ To perform statistical group comparisons across the range of chosen densities, we constructed curves showing the change in measures of interest as a function of the density. Functional data analysis (FDA) was performed with network measures treated as a function of y = *f*(x), allowing summation across densities and obviating the need for multiple testing (inline with^61^) using the GAT software.^48^ One-way ANOVA was used to compare the density function obtained using FDA between the 2 groups, followed by an estimation of the unbiased effect sizes using Hedges *g*.

## Results

### Core Analytic Cohort

The sample comprised 84 patients at first contact with services for an episode of psychosis, with an average age of 29.6 (SD = 9.8) years. After an average of 4 years, 10 (11.9%) were lost to follow-up and 74 (88.1%) were successfully followed up (mean years = 3.7, SD = 1.4). At onset, patients lost to follow-up were more likely to live with others (*x*^2^_(1)_ = 3.88, *P* =.049) and to have a higher GAF symptom score (*t*_(53)_ = 3.08, *P* =.003). There were no other significant differences between those patients who were followed up and those who were lost to follow-up (supplementary table 1). Among patients who were followed up, 4 (5.4%) did not have sufficient information to establish whether they had developed TR. Therefore, the core analytic sample included in subsequent analyses comprised 70 patients with an average age of 28.2 years (SD = 7.3). Of these, 17 (24.3%) met criteria for TR at the end of the follow-up period, while 53 (74.7%) were defined as non-TR.

### Comparisons Between TR and Non-TR Groups

Baseline sociodemographic and clinical characteristics of the TR and non-TR groups are presented in table 1. There were no significant differences between the 2 groups in baseline sociodemographic and clinical characteristics. There were also no differences in age at first contact with services between the TR (mean_years_ = 25.8, SD = 5.4) and non-TR groups (mean_year_ = 28.8, SD = 7.8; *t*_(68)_ = 1.47, *P* =.146).

**Table 1.** Baseline Sample Characteristics for the Non-TR and TR Groups

Baseline Characteristic	Non-TR, *N =* 53 (75.7%)	TR, *N* = 17 (24.3%)	Test Statistics		
	Mean (SD)/*N*(%)	Mean (SD)/*N*(%)	*t/U/x* ^2^	df	*P*
Age_years_	28.8 (7.8)	25.8 (5.4)	1.47	68	.146
Follow-up length_years_	3.5 (1.5) Range = 1–9	4.6 (0.78) Range = 3–6	−3.00	69	.004
Time from starting AP to MRI_days_	40.5 (31.7)	48.3 (37.0)	−0.688		.491
DUP_ weeks_	64.0 (207.8)	50.7 (114.2)	−1.32		.187
*Gender*			0.002	1	.969
Female	12 (23.1)	4 (23.5)			
Male	40 (76.9)	13 (76.5)			
*Ethnicity*			0.44	2	.803
White ethnic groups	19 (36.5)	8 (44.4)			
Black ethnic groups	18 (34.6)	6 (33.3)			
Other	15 (28.9)	4 (22.2)			
*Living arrangements*			1.18	1	.277
Alone	21 (46.7)	10 (62.5)			
Not alone	24 (53.3)	6 (37.5)			
*Relationship status*			3.42	1	.064
Single/separated	32 (71.1)	15 (93.8)			
Stable relationship	13 (28.9)	1 (6.2)			
*Clinical presentation*					
GAF symptoms	48.3 (20.0)	37.1 (12.7)	1.51	63	.138
GAF disability	49.5 (20.0)	55.9 (18.3)	−0.83	63	.412
Positive symptom dimension	−0.28 (1.21)	0.01 (1.37)	−0.78	63	.441
Negative symptom dimension	−0.17 (0.96)	0.36 (0.30)	−1.77	62	.082
Disorganisation dimension	−0.13 (0.61)	−0.01 (0.52)	−0.65	62	.519
Excited symptom dimension	−0.13 (0.42)	−0.17 (0.51)	0.29	63	.770
Depressed symptom dimension	−0.13 (0.61)	−0.05 (0.51)	−0.49	63	.628
*Baseline diagnosis*			1.31	1	.520
Schizophrenia spectrum disorders	38 (71.7)	15 (82.3)			
Affective psychoses	13 (24.5)	2 (11.8)			
Other	2 (3.8)	1 (5.9)			

*Note*: TR, treatment resistant; AP antipsychotic medications; MRI, Magnetic resonance imaging; df, degrees of freedom; DUP, Duration of untreated psychosis; GAF, Global Assessment of Functioning Scale.

### Confirmatory Factor Analysis

The CFA of our core analytic sample produced an excellent fit of the model: CFI = 0.959, RMSEA = 0.052 (90% CI 0.037–0.067), and SRMR = 0.071. There were no significant differences between TR and non-TR groups in these symptom dimensions. Scores for PANSS items the 2 are presented in supplementary table 2.

### Gyrification-Based Connectome and TR

**Fig. 2.**
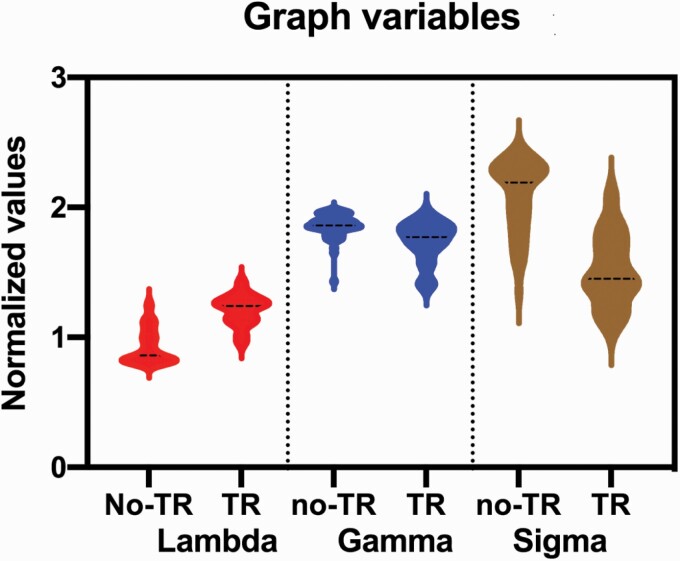
Plot depicting the relationship, adjusted for TR status, age and sex and the positive symptom dimension, between the residuals of small-worldness index (σ) and positive symptom dimension in the entire sample. Also, see supplementary figure 2.

Results from the graph analyses are presented in table 2. Compared to patients in the non-TR group, patients in the TR group had a significant reduction in small-worldness (Hedges’s *g* = 2.09, *P* < .001) and reduced clustering coefficient (Hedges’s *g* = 1.07, *P* < .001) with increased path length (Hedges’s *g* = −2.17, *P* < .001). As shown in figure 2, having adjusted the analyses for age, gender and TR status, the positive symptom dimension was positively correlated with higher small-worldness (*r* = .41, *P* =.001) across the entire sample. This relationship was in the same direction, although not statistically significant, when the correlation was performed in the TR and non-TR groups separately (*r* = .26 to 0.35, *P* = .07–.2; supplementary figure 2). There were no similar correlations with the other symptom dimensions (table 3).

**Table 2.** Graph Variables and Their Effect Sizes in the TR and non-TR Groups

Graph Variables	FDA Mean (SD)		*F* value (*P*-value)	Hedges’ *g*
	Non-TR(*N* = 53)	TR (*N* = 17)		
Path length (λ)	0.93 (0.13)	1.21 (0.12)	60.39 (<.001)	−2.17
Clustering coefficient (ϒ)	1.86 (0.10)	1.74 (0.16)	14.66 (<.001)	1.07
Small-worldness (σ)	2.09 (0.28)	1.51 (0.28)	56.02 (<.001)	2.09

*Note*: TR, treatment resistance; FDA, Functional Data Analysis; all 3 comparisons survived Bonferroni correction for multiple testing (ie, *P* < .016). Also see supplementary figure 1.

**Table 3.** Correlation Between Graph Variables and Symptom Dimensions

	Small-Worldness (σ)	Path Length (λ)	Clustering Coefficient (ϒ)
	*r* (*P*-value)	*r* (*P*-value)	*r* (*P*-value)
Positive dimension	.41 (.001)^a^	−.34 (.006)	.33 (.008)
Negative dimension	−.14 (.30)	.08 (.52)	−.15 (.24)
Disorganized dimension	.20 (.12)	−.18 (.18)	.12 (.34)
Excited symptom dimension	−.19 (.14)	.16 (.21)	.21 (.09)
Depressed symptom dimension	−.038 (.76)	−.23 (.06)	−.07 (.59)

*Note*: Unstandardized residuals adjusted for binary TR status, age and sex. *r* = Pearson correlation coefficient; degree of freedom df = 64 for each correlation.

^a^Survives Bonferroni correction for multiple testing (ie, *P* < .005).

We also undertook a direct comparison of the LGI values of the 148 regions between the TR and non-TR groups, with False Discovery Rate (FDR) corrected at *P* < .05 as threshold for significance. This comparison did not identify any between-group difference, indicating that disruptions in the covariance pattern of gyrification (based on (σ, ϒ, and λ) are more pronounced than any subtle difference in absolute regional cortical folding. The uncorrected comparisons of LGI values for the 148 regions are presented in supplementary table 3.

## Discussion

To our knowledge, this is the first study to have examined the value of gyrification at illness onset as a predictor of TR occurring over the subsequent 4 years. Our results point to an association between a reduced coordination of processes driving cortical folding at the whole brain level (σ), stemming from both a neighborhood-level disruption (λ) and a reduced distributed relationship (ϒ), and the onset of TR. Thus, patients with first-episode psychosis who later developed TR, already displayed, at first presentation, a significant aberration in cortex-wide covariance of folding patterns when compared to patients who do not go on to develop TR. This indicates that the development of TR is likely related to the maturational coordination of the entire cortex, rather than to the degree of folding of any single region.

While structural covariance has not been examined in relation to prospectively determined TR to date, a recent study reported that thickness-based group-level structural covariance was not altered at whole brain level in patients with chronic schizophrenia resistant to treatment; instead, covariance was only higher among those regions that showed reduced thickness in TR.^62^ In contrast, here we report an overall reduction in gyrification-based, perturbation-derived patient-level structural covariance in relation to TR. While thickness-based covariance has been interpreted as an index of intra-cortical connectivity,^62^ gyrification-based covariance is best interpreted as an index of synchronized early maturation, given the in-utero synchronization of sulcal development.^63^ Thus, the reduction of small-worldness in perturbation-based LGI networks probably indicates a loss of coordination in cortical folding at the whole brain level, likely occurring during early development.

In contrast to our previous observations when contrasting short-term (12-week) responders with non-responders,^27^ we did not observe localized differences in gyrification between patients who later developed TR and those who did not. This supports the prevailing notion that TR is unlikely to be related to specific neuroanatomical defects.^64,65^ Instead, it may rather relate to a diffuse but subtle neurodevelopmental aberration that occurs either in utero or in early life. Consequently, it is likely that we observe no localizable effects, and that instead disrupted maturational relationships (covariance) among brain regions result in abnormal network-level topology. Here, we observe reduced regional segregation (clustering) and reduced overall integration of the morphological connectome, resulting in reduced small-worldness in covariance. Given the major role played by axonal tension in the formation of cortical folds,^66,67^ we surmise that TR results from a weakening of axonal tensions that arise from reduced inter-regional connectivity in the neonatal brain. As a result, the folding of spatially proximal, physically connected brain regions may not be synchronized and result in reduced clustering. Although the large effect size changes we find indicates >90% chance^68^ that a randomly selected patient with first-episode psychosis could be correctly identified to come from the TR group based on the reduced small-worldness of their cortical morphology, it remains unclear how well this prediction would perform at a single patient level.

Intriguingly, we found higher small-worldness was correlated with higher levels of positive symptoms at illness onset, and that small-worldness was reduced among patients with TR. Some evidence on the neurobiology of TR suggests that patients with TR may have a normodopaminergic/hyperglutamatergic status, while a predominantly hyperdopaminergic/normoglutamatergic status (ie, increased presynaptic dopamine) could relate to a higher degree of treatment responsiveness.^3,69–71^ Consistent with these suggestions, we speculate that a substantial number of individuals with a predominantly hyperdopaminergic pattern and higher positive symptoms at presentation may not have a notable cortical maturational deficit resulting in increased small-worldness of gyrification networks. However, those with less maturational coordination of cortical folding may go on to exhibit TR. The case for a neurodevelopmental characterization of TR is further strengthened by the observation that a younger age of onset,^72^ low IQ and family history of schizophrenia are all associated with TR.^73^ Prospectively designed hybrid PET/MR and glutamate MRS studies would help test the notion that a neurodevelopmental subtype with predominant glutamatergic deficits is specifically associated with TR.

It is noteworthy that network methods used in previous cross-sectional structural covariance studies also relied on group-based correlation,^74^ but the edges of those networks represented population-level (ie, between-subjects) relationships among regional gyrification indices. In contrast, the edges derived from our approach using deviance matrices represent the estimated relationship at an individual level (within-subject). Thus, the topological metrics from our network approach, based on second-order statistics (distance vs. correlation coefficient), are likely idiographic. The edges between 2 regions (nodes) in the individual gyrification networks do not imply direct connectivity but represent the absolute contribution each individual makes to the observed group-specific coordination in gyrification.

### Methodological Considerations

The strengths of this study include the evaluation of brain morphology at the time of the first presentation to services, and the prospective evaluation of illness course over the first 4 years of illness. We have examined the onset of TR from the time of first contact with mental health services. The factor model of psychosis symptoms we used was derived from previous studies^56^ and shown to be optimal for the evaluation of patients with first-episode psychosis.^75–77^ The symptom dimensions were based on the PANSS, which has been shown to be resilient to the effects of age, chronicity of illness^78^ and short-term medication withdrawal.^79^ As the MRI data were obtained at the first-episode, before the evolution to TR status, the findings are not likely to be confounded by chronicity of illness or prolonged exposure to medications.

Although our length of DUP was consistent with that of other cohorts of first-episode psychoses,^80–82^ it was still shorter than that reported in some other studies.^83–85^ Given the variability in the definitions of DUP,^86^ we urge caution when generalizing our observations to all studies of first-episode psychosis. It is also possible that some patients in the non-TR group could have been TR but were unable did not accept or tolerate clozapine; similarly, it is also possible that some individuals in the non-TR group could have developed TR if they were followed up over a longer time period of time. However, we believe this latter possibility is unlikely, as we have previously shown that in most cases, TR becomes apparent early in the course of illness. Although evidence suggests there may be an early and a late treatment-resistant subtype,^4,5^ we did not have a sufficient number of patients to investigate if gyrification can discriminate between them. Similarly, it is feasible that a relatively small and unbalanced number of patients could have had an impact on our results. Nonetheless, our sample size is consistent with that of previous reports,^3,16,22,25^ and the definition of TR we used took into account instances when antipsychotics were stopped due to side effects rather than lack of improvement, thus ensuring it reflected true TR. Finally, the lack of a healthy control group limits our ability to comment on whether the patterns seen in non-TR individuals are “deviant” from those of individuals without a diagnosis of psychosis. As we now know what are the demographic and socioeconomic profiles of patients that are likely to develop TR over 4 years, we anticipate undertaking future matched data-collection with cohorts of healthy subjects that match the predicted non-TR and TR groups.

### Conclusion

Several putative mechanistic pathways that ultimately result in treatment resistance in psychotic disorders may converge on disruption of coordinated cortical maturation. Our observations raise the possibility of defining a therapeutically meaningful “neurotype” of psychosis based on an easily accessible structural imaging assay of cortical folding patterns.

## Supplementary Material

sbab035_suppl_Supplementary_Materials
