# Supplementary material for: Structural Covariance of Cortical Gyrification at Illness Onset in Treatment Resistance: A Longitudinal Study of First-Episode Psychoses
Source: Schizophr Bull. 2021 Apr 14;47(6):1729–39. doi: 10.1093/schbul/sbab035 (PMC8530394; doi:10.1093/schbul/sbab035)

**Supplementary material**

**MRI acquisition.** The MRI scans were obtained as soon as possible after first contact with psychiatric service, whenever deemed appropriate by the treating clinician, to ensure minimal exposure to antipsychotic medications in patients. All MRI scans were acquired in a 3-T Signa HDx scanner (General Electric) at the Centre for Neuroimaging Sciences, Institute of Psychiatry, London, England. A sagittal 3-dimensional magnetization- prepared rapid-acquisition gradient-echo volumetric scan was obtained from each subject. The scan had an image matrix size of 256 × 256 × 166 voxels, with an in-plane voxel size of 1.02 × 1.02 mm and a slice thickness of 1.2 mm (echo time, 2.848 milliseconds; repetition time, 6.988 milliseconds; inversion time, 650 milliseconds; excitation flip angle, 20°; 1 data average).

**Image inspection criteria used for quality checking.** We adopted an approach of careful quality control to remove poor quality data affected by movement artifacts which increase the need for manual editing using control points. We did not introduce manually placed control points, but the intermediate output files with grey-white boundary definitions were inspected for each subject, in a blind fashion by a single rater, before further analysis was carried out. We eventually excluded 4 scans (1 control, 1 non-TR and 2 TR). The image inspection criteria we have employed for gyrification analysis is summarised below.

*Exclusion criteria for motion artifacts:*

Scans with at least 1 of the following 3 criteria were excluded owing to motion artifacts:

1. Images too grainy: grey–white matter boundary is clearly invisible in more than 2 anatomically distinct regions
2. Significant edge ringing artifacts: more than 2 rings noted with associated blurring of grey–white matter boundary in more than 2 anatomically distinct regions
3. Less severe motion artifacts/grainy image but not satisfying criteria 1 and 2, but either

- fails Freesurfer cortical reconstruction owing to substantial topological defects; or
- presence of more than 2 handles/holes that require manual intervention (e.g., hole-filling, defining control points, removal of obscure/uncertain pia-like tissue) to define grey–white matter boundaries despite Freesurfer’s automatic topological fixation procedure

**Surface Extraction**. Surface extraction and cortical parcellation were carried outwithFreeSurferversion4.5.0;^1^ after skull-stripping and intensity correction, the gray–white matter boundary for each cortical hemisphere was determined with tissue intensity and neighbourhood constraints. The resulting surface boundary was tessellated to generate multiple vertices across the whole brain before inflating. All surfaceswere visually inspected after an automated topology fixation procedure, and remaining minor defects were manually corrected as recommended by the software guidelines. The expansion of the resulting gray–white interface created the pial surface with a point-to-point correspondence followed by spherical morphing and spherical registration. The parcellations were obtained by inverting spherical morphing procedure to map back the average spherical representation onto the inflated surface of each subject. The parcellations were obtained with Destrieux sulcogyral-based atlas, which follows the anatomical conventions of Duvernoy. The six combined sulcogyral regions selected a priori include front marginal region; orbital frontal region (including H-shaped orbital sulcus); inferior, middle, and frontal regions (both gyri and sulci); and frontal pole (comprising transverse frontopolar gyrusand sulcus). Anatomical boundaries of each individual region are described by Destrieuxet al.^2^. Figure S1 in Supplement 1 dis-plays the selected sulcogyral regions.

**Graph-based metrics**

Measures of integration, segregation and small-worldness were derived from each group-specific graph whose edges were constructed on the basis of group-level correlation among the individual brain regions (nodes).

(1) *Integration*: Shortest path length L_p_ between two regions (A, B) that refers to the minimum number of connections that link A and B. If A and B have direct structural covariance, then they will have a direct connection in the gyrification connectome, with their L_p_ being 1. If A and B do not have direct covariance, but if A covaries with C, and C covaries with B, then the L_p_ between A and B will be 2 (mediated by 2 connections: AC and CB). The average shortest path length between all pairs of regions in the network gives the characteristic path length of the network (ML_p_).

(2) *Segregation*: Clustering coefficient C_p_ indicates the presence of a high degree of covariance (number of connections or edges) among neighbouring regions. The average of clustering coefficients of each region (or node) provides the clustering coefficient of the network (MC_p_).

(3) In line with previous connectomic studies, we estimated the small-world index by comparing the estimated topological properties (clustering coefficient = MC_p_, and characteristic path length = ML_p_) of the networks with corresponding mean values of null random graphs (MC_null_ and ML_null_ of null networks) constructed with same number of nodes, edges and degree distribution as the gyrification based networks. Small world index (sigma) is given by (sigma=gamma/lambda = [MC_p_/MC_null_]/[ML_p_/ ML_null_]). A sigma>1 suggests a small-world network that has a relatively high segregation and integration compared to random null networks.^3^ Most biological networks, including neuroimaging based connectomes show sigma>1, indicating efficient information transfer at a relatively low wiring cost.

**References:**

**1.** Fischl B, Dale AM. Measuring the thickness of the human cerebral cortex from magnetic resonance images. *Proc Natl Acad Sci U S A* Sep 26 2000;97(20):11050-11055.

**2.** Destrieux C, Fischl B, Dale A, Halgren E. Automatic parcellation of human cortical gyri and sulci using standard anatomical nomenclature. *Neuroimage* Oct 15 2010;53(1):1-15.

**3.** Humphries MD, Gurney K. Network 'small-world-ness': a quantitative method for determining canonical network equivalence. *PLoS One* Apr 30 2008;3(4):e0002051.

**Supplementary Table 1.** Baseline sample characteristics for those patients who were followed up and those who were lost to follow up

| Baseline characteristic | | Lost to follow up  N=10 (11.9%) | Followed up  N=74 (88.1%) | Test Statistics | | |
| --- | --- | --- | --- | --- | --- | --- |
|  | | Mean (SD)/N(%) | Mean (SD)/N(%) | t/U/x^2^ | df | *P* |
|  | |  |  |  |  |  |
| DUP _days_ Mean (SD) | | 60.2 (68.5) | 59.7 (181.7) | 1.689 |  | .091 |
|  | |  |  |  |  |  |
| Age _years_ | | 31.0 (12.4) | 28.2 (7.3) | 0.963 | 80 | .338 |
|  | |  |  |  |  |  |
| *Gender* | |  |  | 0.004 | 1 | .950 |
|  | Female | 2 (25.0) | 19 (26.0) |  |  |  |
|  | Male | 6 (75.0) | 54 (74.0) |  |  |  |
|  |  |  |  |  |  |  |
| *Ethnicity* | |  |  | 0.365 | 2 | .833 |
|  | White ethnic groups | 4 (50.0) | 29 (39.2) |  |  |  |
|  | Black ethnic groups | 2 (25.0) | 24 (32.4) |  |  |  |
|  | Other | 2 (25.0) | 21 (28.4) |  |  |  |
|  |  |  |  |  |  |  |
| *Living arrangements* | |  |  | 3.880 | 1 | .049 |
|  | Alone | 1 (12.5) | 32 (49.2) |  |  |  |
|  | Not alone | 7 (87.5) | 33 (50.8) |  |  |  |
|  |  |  |  |  |  |  |
| *Relationship status* | |  |  | 2.738 | 1 | .099 |
|  | Single/separated | 8 (100) | 48 (73.9) |  |  |  |
|  | Stable relationship | - | 17 (26.1) |  |  |  |
|  |  |  |  |  |  |  |
| *Clinical presentation* | |  |  |  |  |  |
|  | GAF symptoms | 58.7 (17.2) | 41.5 (18.8) | 3.08 | 53 | .003 |
|  | GAF disability | 58.2 (19.1) | 52.3 (14.8) | 1.02 | 53 | .311 |

TR, treatment resistance; SD, standard deviation; df, degrees of freedom; DUP, Duration of untreated psychosis; GAF, Global Assessment of Functioning Scale

**Supplementary Table 2.** Presents distribution of each PANSS item in TR and non-TR groups

|  | **Total (N=70)** | **non-TR (N=53)** | **TR (N=17)** |
| --- | --- | --- | --- |
| PANSS items | Mean (SD) | Mean (SD) | Mean (SD) |
| ***Positive symptoms*** |  |  |  |
| Delusions | 2.8 (1.7) | 2.7 (1.6) | 3.1 (1.9) |
| Conceptual disorganisation | 1.8 (0.90) | 1.8 (0.92) | 2.0 (0.93) |
| Hallucinatory behaviour | 2.5 (1.7) | 2.5 (1.7) | 2.8 (1.9) |
| Excitement | 1.5 (0.99) | 1.6 (1.1) | 1.1 (0.26) |
| Grandiosity | 1.7 (1.3) | 1.8 (1.3) | 1.7 (1.6) |
| Suspiciousness/persecution | 2.3 (1.5) | 2.5 (1.5) | 2.0 (1.5) |
| Hostility | 1.4 (0.77) | 1.4 (0.76) | 1.3 (0.90) |
|  |  |  |  |
| ***Negative symptoms*** |  |  |  |
| Blunted affect | 2.3 (1.6) | 2.1 (1.5) | 3.4 (1.7) |
| Emotional withdrawal | 2.2 (1.2) | 2.1 (1.2) | 2.6 (1.3) |
| Poor rapport | 1.8 (1.0) | 1.6 (0.95) | 2.2 (1.3) |
| Passive/apathetic social withdrawal | 2.5 (1.6) | 2.6 (1.7) | 2.6 (1.7) |
| Difficulty in abstract thinking | 2.4 (1.4) | 2.3 (1.4) | 2.7 (1.3) |
| Lack of spontaneity & flow of conversation | 2.2 (1.5) | 2.1 (1.4) | 2.9 (1.7) |
| Stereotyped thinking | 1.6 (0.97) | 1.6 (0.97) | 1.7 (0.88) |
|  |  |  |  |
| ***General psychopathology*** |  |  |  |
| Somatic concern | 1.6 (0.93) | 1.6 (0.91) | 1.9 (1.1) |
| Anxiety | 2.5 (1.1) | 2.5 (1.2) | 2.6 (0.91) |
| Guilt feelings | 1.9 (1.3) | 2.0 (1.3) | 1.7 (1.2) |
| Tension | 1.6 (0.84) | 1.6 (0.85) | 1.6 (0.83) |
| Mannerisms & posturing | 1.2 (0.50) | 1.2 (0.56) | 1.1 (0.26) |
| Depression | 2.6 (1.5) | 2.6 (1.6) | 2.7 (1.3) |
| Motor retardation | 1.6 (1.0) | 1.5 (0.99) | 1.9 (1.1) |
| Uncooperativeness | 1.2 (0.55) | 1.3 (0.56) | 1.2 (0.56) |
| Unusual thought content | 1.8 (1.2) | 1.8 (1.2) | 1.9 (1.1) |
| Disorientation | 1.3 (0.57) | 1.3 (0.56) | 1.3 (0.62) |
| Poor attention | 1.5 (0.85) | 1.5 (0.89) | 1.5 (0.83) |
| Lack of judgement & insight | 3.0 (1.7) | 2.8 (1.6) | 3.5 (2.1) |
| Disturbance of volition | 1.5 (0.86) | 1.5 (0.80) | 1.6 (1.1) |
| Poor impulse control | 1.3 (0.82) | 1.3 (0.68) | 1.3 (1.1) |
| Preoccupation | 2.0 (1.2) | 1.9 (1.1) | 2.1 (1.4) |
| Active social avoidance | 2.3 (1.4) | 2.5 (1.5) | 2.1 (1.4) |

TR, treatment resistance; SD, standard deviation; PANSS, Positive and Negative Syndrome Scale

**Supplementary Table 3.** The uncorrected comparison results of LGI values of the 148 regions

|  |  | **Levene's Test for Equality of Variances** | | **t-test for Equality of Means** | | | | | |  |
| --- | --- | --- | --- | --- | --- | --- | --- | --- | --- | --- |
|  |  | **F** | **Sig.** | **t** | **df** | **Sig. (2-tailed)** | **Mean Difference** | **Std. Error Difference** | **95%CI** |  |
| **lh_G_and_S_frontomargin_LGI** | Equal variances assumed | 0.858 | 0.357 | -0.817 | 68 | 0.417 | -0.02889 | 0.03539 | -0.09950 | 0.04172 |
| **lh_G_and_S_frontomargin_LGI** | Equal variances not assumed |  |  | -0.936 | 35.035 | 0.356 | -0.02889 | 0.03088 | -0.09158 | 0.03379 |
| **lh_G_and_S_occipital_inf_LGI** | Equal variances assumed | 1.414 | 0.239 | -0.195 | 68 | 0.846 | -0.00909 | 0.04650 | -0.10188 | 0.08370 |
| **lh_G_and_S_occipital_inf_LGI** | Equal variances not assumed |  |  | -0.229 | 36.798 | 0.820 | -0.00909 | 0.03969 | -0.08953 | 0.07135 |
| **lh_G_and_S_paracentral_LGI** | Equal variances assumed | 1.256 | 0.266 | -1.774 | 68 | 0.081 | -0.05489 | 0.03095 | -0.11665 | 0.00687 |
| **lh_G_and_S_paracentral_LGI** | Equal variances not assumed |  |  | -1.972 | 32.853 | 0.057 | -0.05489 | 0.02784 | -0.11154 | 0.00176 |
| **lh_G_and_S_subcentral_LGI** | Equal variances assumed | 0.196 | 0.659 | -0.182 | 68 | 0.856 | -0.01961 | 0.10784 | -0.23481 | 0.19558 |
| **lh_G_and_S_subcentral_LGI** | Equal variances not assumed |  |  | -0.186 | 28.044 | 0.854 | -0.01961 | 0.10554 | -0.23579 | 0.19656 |
| **lh_G_and_S_transv_frontopol_LGI** | Equal variances assumed | 0.161 | 0.689 | -0.652 | 68 | 0.516 | -0.02042 | 0.03131 | -0.08289 | 0.04205 |
| **lh_G_and_S_transv_frontopol_LGI** | Equal variances not assumed |  |  | -0.676 | 28.710 | 0.505 | -0.02042 | 0.03023 | -0.08227 | 0.04142 |
| **lh_G_and_S_cingul-Ant_LGI** | Equal variances assumed | 0.103 | 0.749 | -0.754 | 68 | 0.453 | -0.02315 | 0.03069 | -0.08439 | 0.03810 |
| **lh_G_and_S_cingul-Ant_LGI** | Equal variances not assumed |  |  | -0.740 | 26.279 | 0.466 | -0.02315 | 0.03126 | -0.08736 | 0.04107 |
| **lh_G_and_S_cingul-Mid-Ant_LGI** | Equal variances assumed | 1.102 | 0.297 | 0.024 | 68 | 0.981 | 0.00067 | 0.02752 | -0.05423 | 0.05558 |
| **lh_G_and_S_cingul-Mid-Ant_LGI** | Equal variances not assumed |  |  | 0.022 | 23.304 | 0.983 | 0.00067 | 0.03050 | -0.06237 | 0.06372 |
| **lh_G_and_S_cingul-Mid-Post_LGI** | Equal variances assumed | 1.797 | 0.185 | -0.099 | 68 | 0.921 | -0.00310 | 0.03129 | -0.06555 | 0.05934 |
| **lh_G_and_S_cingul-Mid-Post_LGI** | Equal variances not assumed |  |  | -0.085 | 21.852 | 0.933 | -0.00310 | 0.03659 | -0.07901 | 0.07280 |
| **lh_G_cingul-Post-dorsal_LGI** | Equal variances assumed | 0.001 | 0.977 | -0.073 | 68 | 0.942 | -0.00394 | 0.05390 | -0.11150 | 0.10362 |
| **lh_G_cingul-Post-dorsal_LGI** | Equal variances not assumed |  |  | -0.067 | 23.657 | 0.947 | -0.00394 | 0.05906 | -0.12594 | 0.11806 |
| **lh_G_cingul-Post-ventral_LGI** | Equal variances assumed | 2.409 | 0.125 | 1.050 | 68 | 0.297 | 0.04868 | 0.04636 | -0.04384 | 0.14119 |
| **lh_G_cingul-Post-ventral_LGI** | Equal variances not assumed |  |  | 1.348 | 46.011 | 0.184 | 0.04868 | 0.03611 | -0.02400 | 0.12135 |
| **lh_G_cuneus_LGI** | Equal variances assumed | 0.490 | 0.486 | -0.480 | 68 | 0.633 | -0.02698 | 0.05625 | -0.13922 | 0.08526 |
| **lh_G_cuneus_LGI** | Equal variances not assumed |  |  | -0.467 | 25.951 | 0.644 | -0.02698 | 0.05775 | -0.14570 | 0.09174 |
| **lh_G_front_inf-Opercular_LGI** | Equal variances assumed | 0.389 | 0.535 | -0.390 | 68 | 0.697 | -0.04602 | 0.11785 | -0.28119 | 0.18915 |
| **lh_G_front_inf-Opercular_LGI** | Equal variances not assumed |  |  | -0.368 | 24.711 | 0.716 | -0.04602 | 0.12509 | -0.30379 | 0.21176 |
| **lh_G_front_inf-Orbital_LGI** | Equal variances assumed | 0.010 | 0.922 | -0.438 | 68 | 0.663 | -0.05054 | 0.11531 | -0.28065 | 0.17956 |
| **lh_G_front_inf-Orbital_LGI** | Equal variances not assumed |  |  | -0.450 | 28.231 | 0.656 | -0.05054 | 0.11242 | -0.28073 | 0.17965 |
| **lh_G_front_inf-Triangul_LGI** | Equal variances assumed | 1.722 | 0.194 | 0.512 | 68 | 0.610 | 0.04581 | 0.08950 | -0.13278 | 0.22439 |
| **lh_G_front_inf-Triangul_LGI** | Equal variances not assumed |  |  | 0.593 | 35.931 | 0.557 | 0.04581 | 0.07721 | -0.11078 | 0.20240 |
| **lh_G_front_middle_LGI** | Equal variances assumed | 0.092 | 0.762 | -1.003 | 68 | 0.320 | -0.04968 | 0.04955 | -0.14856 | 0.04919 |
| **lh_G_front_middle_LGI** | Equal variances not assumed |  |  | -1.075 | 30.589 | 0.291 | -0.04968 | 0.04621 | -0.14398 | 0.04462 |
| **lh_G_front_sup_LGI** | Equal variances assumed | 0.934 | 0.337 | -0.902 | 68 | 0.370 | -0.02487 | 0.02758 | -0.07990 | 0.03017 |
| **lh_G_front_sup_LGI** | Equal variances not assumed |  |  | -0.832 | 24.026 | 0.413 | -0.02487 | 0.02987 | -0.08651 | 0.03678 |
| **lh_G_Ins_lg_and_S_cent_ins_LGI** | Equal variances assumed | 0.132 | 0.717 | -0.166 | 68 | 0.869 | -0.02012 | 0.12110 | -0.26177 | 0.22152 |
| **lh_G_Ins_lg_and_S_cent_ins_LGI** | Equal variances not assumed |  |  | -0.163 | 26.329 | 0.871 | -0.02012 | 0.12318 | -0.27316 | 0.23291 |
| **lh_G_insular_short_LGI** | Equal variances assumed | 0.269 | 0.606 | -0.419 | 68 | 0.677 | -0.04991 | 0.11915 | -0.28768 | 0.18786 |
| **lh_G_insular_short_LGI** | Equal variances not assumed |  |  | -0.400 | 25.163 | 0.693 | -0.04991 | 0.12489 | -0.30703 | 0.20721 |
| **lh_G_occipital_middle_LGI** | Equal variances assumed | 0.225 | 0.637 | -0.157 | 68 | 0.876 | -0.00844 | 0.05383 | -0.11586 | 0.09898 |
| **lh_G_occipital_middle_LGI** | Equal variances not assumed |  |  | -0.171 | 31.592 | 0.865 | -0.00844 | 0.04938 | -0.10907 | 0.09219 |
| **lh_G_occipital_sup_LGI** | Equal variances assumed | 0.228 | 0.635 | -0.445 | 68 | 0.658 | -0.02180 | 0.04905 | -0.11968 | 0.07608 |
| **lh_G_occipital_sup_LGI** | Equal variances not assumed |  |  | -0.421 | 24.870 | 0.678 | -0.02180 | 0.05183 | -0.12858 | 0.08497 |
| **lh_G_oc-temp_lat-fusifor_LGI** | Equal variances assumed | 0.034 | 0.855 | 0.284 | 68 | 0.778 | 0.00877 | 0.03092 | -0.05293 | 0.07047 |
| **lh_G_oc-temp_lat-fusifor_LGI** | Equal variances not assumed |  |  | 0.293 | 28.503 | 0.772 | 0.00877 | 0.02998 | -0.05259 | 0.07012 |
| **lh_G_oc-temp_med-Lingual_LGI** | Equal variances assumed | 0.071 | 0.791 | 0.284 | 68 | 0.778 | 0.01160 | 0.04090 | -0.07001 | 0.09322 |
| **lh_G_oc-temp_med-Lingual_LGI** | Equal variances not assumed |  |  | 0.295 | 28.956 | 0.770 | 0.01160 | 0.03930 | -0.06878 | 0.09198 |
| **lh_G_oc-temp_med-Parahip_LGI** | Equal variances assumed | 0.204 | 0.653 | 0.704 | 68 | 0.484 | 0.02606 | 0.03700 | -0.04777 | 0.09990 |
| **lh_G_oc-temp_med-Parahip_LGI** | Equal variances not assumed |  |  | 0.734 | 29.024 | 0.469 | 0.02606 | 0.03551 | -0.04655 | 0.09868 |
| **lh_G_orbital_LGI** | Equal variances assumed | 0.000 | 0.991 | -0.447 | 68 | 0.656 | -0.01805 | 0.04039 | -0.09865 | 0.06254 |
| **lh_G_orbital_LGI** | Equal variances not assumed |  |  | -0.465 | 28.960 | 0.645 | -0.01805 | 0.03881 | -0.09742 | 0.06132 |
| **lh_G_pariet_inf-Angular_LGI** | Equal variances assumed | 0.210 | 0.648 | -0.923 | 68 | 0.359 | -0.04320 | 0.04682 | -0.13663 | 0.05023 |
| **lh_G_pariet_inf-Angular_LGI** | Equal variances not assumed |  |  | -0.992 | 30.726 | 0.329 | -0.04320 | 0.04356 | -0.13208 | 0.04568 |
| **lh_G_pariet_inf-Supramar_LGI** | Equal variances assumed | 0.106 | 0.746 | -0.755 | 68 | 0.453 | -0.04897 | 0.06483 | -0.17834 | 0.08040 |
| **lh_G_pariet_inf-Supramar_LGI** | Equal variances not assumed |  |  | -0.820 | 31.303 | 0.419 | -0.04897 | 0.05974 | -0.17077 | 0.07283 |
| **lh_G_parietal_sup_LGI** | Equal variances assumed | 0.097 | 0.757 | -1.584 | 68 | 0.118 | -0.07475 | 0.04718 | -0.16889 | 0.01939 |
| **lh_G_parietal_sup_LGI** | Equal variances not assumed |  |  | -1.687 | 30.178 | 0.102 | -0.07475 | 0.04431 | -0.16523 | 0.01573 |
| **lh_G_postcentral_LGI** | Equal variances assumed | 0.105 | 0.747 | -1.353 | 68 | 0.181 | -0.07457 | 0.05512 | -0.18456 | 0.03543 |
| **lh_G_postcentral_LGI** | Equal variances not assumed |  |  | -1.522 | 33.668 | 0.137 | -0.07457 | 0.04900 | -0.17419 | 0.02506 |
| **lh_G_precentral_LGI** | Equal variances assumed | 0.137 | 0.712 | -1.405 | 68 | 0.165 | -0.08589 | 0.06114 | -0.20789 | 0.03610 |
| **lh_G_precentral_LGI** | Equal variances not assumed |  |  | -1.431 | 27.908 | 0.163 | -0.08589 | 0.06000 | -0.20882 | 0.03704 |
| **lh_G_precuneus_LGI** | Equal variances assumed | 0.108 | 0.743 | -0.521 | 68 | 0.604 | -0.02530 | 0.04852 | -0.12212 | 0.07152 |
| **lh_G_precuneus_LGI** | Equal variances not assumed |  |  | -0.501 | 25.463 | 0.620 | -0.02530 | 0.05045 | -0.12910 | 0.07851 |
| **lh_G_rectus_LGI** | Equal variances assumed | 2.067 | 0.155 | -0.322 | 68 | 0.748 | -0.00966 | 0.03001 | -0.06956 | 0.05023 |
| **lh_G_rectus_LGI** | Equal variances not assumed |  |  | -0.396 | 41.297 | 0.694 | -0.00966 | 0.02440 | -0.05893 | 0.03960 |
| **lh_G_subcallosal_LGI** | Equal variances assumed | 0.001 | 0.977 | 0.376 | 68 | 0.708 | 0.01499 | 0.03986 | -0.06454 | 0.09453 |
| **lh_G_subcallosal_LGI** | Equal variances not assumed |  |  | 0.394 | 29.326 | 0.696 | 0.01499 | 0.03803 | -0.06275 | 0.09273 |
| **lh_G_temp_sup-G_T_transv_LGI** | Equal variances assumed | 0.249 | 0.619 | -0.292 | 68 | 0.771 | -0.03335 | 0.11427 | -0.26136 | 0.19467 |
| **lh_G_temp_sup-G_T_transv_LGI** | Equal variances not assumed |  |  | -0.294 | 27.342 | 0.771 | -0.03335 | 0.11353 | -0.26616 | 0.19947 |
| **lh_G_temp_sup-Lateral_LGI** | Equal variances assumed | 0.010 | 0.921 | -0.301 | 68 | 0.764 | -0.02498 | 0.08294 | -0.19049 | 0.14053 |
| **lh_G_temp_sup-Lateral_LGI** | Equal variances not assumed |  |  | -0.321 | 30.287 | 0.750 | -0.02498 | 0.07776 | -0.18373 | 0.13377 |
| **lh_G_temp_sup-Plan_polar_LGI** | Equal variances assumed | 2.438 | 0.123 | -0.020 | 68 | 0.984 | -0.00185 | 0.09235 | -0.18614 | 0.18243 |
| **lh_G_temp_sup-Plan_polar_LGI** | Equal variances not assumed |  |  | -0.024 | 39.953 | 0.981 | -0.00185 | 0.07611 | -0.15568 | 0.15197 |
| **lh_G_temp_sup-Plan_tempo_LGI** | Equal variances assumed | 0.530 | 0.469 | -0.601 | 68 | 0.550 | -0.04477 | 0.07452 | -0.19347 | 0.10393 |
| **lh_G_temp_sup-Plan_tempo_LGI** | Equal variances not assumed |  |  | -0.650 | 31.080 | 0.521 | -0.04477 | 0.06892 | -0.18533 | 0.09578 |
| **lh_G_temporal_inf_LGI** | Equal variances assumed | 0.488 | 0.487 | -0.605 | 68 | 0.547 | -0.02407 | 0.03979 | -0.10347 | 0.05533 |
| **lh_G_temporal_inf_LGI** | Equal variances not assumed |  |  | -0.542 | 23.122 | 0.593 | -0.02407 | 0.04438 | -0.11585 | 0.06771 |
| **lh_G_temporal_middle_LGI** | Equal variances assumed | 0.318 | 0.574 | -0.382 | 68 | 0.704 | -0.02553 | 0.06691 | -0.15904 | 0.10798 |
| **lh_G_temporal_middle_LGI** | Equal variances not assumed |  |  | -0.371 | 25.841 | 0.714 | -0.02553 | 0.06889 | -0.16717 | 0.11611 |
| **lh_Lat_Fis-ant-Horizont_LGI** | Equal variances assumed | 0.881 | 0.351 | -0.271 | 68 | 0.787 | -0.03223 | 0.11882 | -0.26933 | 0.20488 |
| **lh_Lat_Fis-ant-Horizont_LGI** | Equal variances not assumed |  |  | -0.253 | 24.359 | 0.802 | -0.03223 | 0.12742 | -0.29499 | 0.23054 |
| **lh_Lat_Fis-ant-Vertical_LGI** | Equal variances assumed | 0.205 | 0.652 | 0.029 | 68 | 0.977 | 0.00337 | 0.11688 | -0.22986 | 0.23660 |
| **lh_Lat_Fis-ant-Vertical_LGI** | Equal variances not assumed |  |  | 0.027 | 24.468 | 0.979 | 0.00337 | 0.12493 | -0.25421 | 0.26096 |
| **lh_Lat_Fis-post_LGI** | Equal variances assumed | 0.003 | 0.960 | -0.063 | 68 | 0.950 | -0.00570 | 0.09091 | -0.18710 | 0.17571 |
| **lh_Lat_Fis-post_LGI** | Equal variances not assumed |  |  | -0.064 | 27.701 | 0.950 | -0.00570 | 0.08962 | -0.18936 | 0.17797 |
| **lh_Pole_occipital_LGI** | Equal variances assumed | 1.770 | 0.188 | -0.988 | 68 | 0.327 | -0.03627 | 0.03670 | -0.10951 | 0.03697 |
| **lh_Pole_occipital_LGI** | Equal variances not assumed |  |  | -1.122 | 34.323 | 0.270 | -0.03627 | 0.03233 | -0.10195 | 0.02942 |
| **lh_Pole_temporal_LGI** | Equal variances assumed | 0.685 | 0.411 | -0.162 | 68 | 0.872 | -0.00547 | 0.03385 | -0.07301 | 0.06208 |
| **lh_Pole_temporal_LGI** | Equal variances not assumed |  |  | -0.167 | 28.578 | 0.869 | -0.00547 | 0.03277 | -0.07253 | 0.06160 |
| **lh_S_calcarine_LGI** | Equal variances assumed | 0.451 | 0.504 | 0.396 | 68 | 0.693 | 0.02199 | 0.05553 | -0.08883 | 0.13281 |
| **lh_S_calcarine_LGI** | Equal variances not assumed |  |  | 0.424 | 30.513 | 0.675 | 0.02199 | 0.05186 | -0.08385 | 0.12783 |
| **lh_S_central_LGI** | Equal variances assumed | 0.247 | 0.621 | -1.260 | 68 | 0.212 | -0.07449 | 0.05911 | -0.19245 | 0.04346 |
| **lh_S_central_LGI** | Equal variances not assumed |  |  | -1.471 | 36.524 | 0.150 | -0.07449 | 0.05062 | -0.17711 | 0.02813 |
| **lh_S_cingul-Marginalis_LGI** | Equal variances assumed | 0.809 | 0.372 | 0.121 | 68 | 0.904 | 0.00430 | 0.03570 | -0.06693 | 0.07554 |
| **lh_S_cingul-Marginalis_LGI** | Equal variances not assumed |  |  | 0.113 | 24.582 | 0.911 | 0.00430 | 0.03803 | -0.07409 | 0.08270 |
| **lh_S_circular_insula_ant_LGI** | Equal variances assumed | 0.320 | 0.573 | -0.548 | 68 | 0.585 | -0.06306 | 0.11498 | -0.29249 | 0.16637 |
| **lh_S_circular_insula_ant_LGI** | Equal variances not assumed |  |  | -0.514 | 24.506 | 0.612 | -0.06306 | 0.12276 | -0.31614 | 0.19003 |
| **lh_S_circular_insula_inf_LGI** | Equal variances assumed | 0.402 | 0.528 | 0.073 | 68 | 0.942 | 0.00831 | 0.11317 | -0.21751 | 0.23413 |
| **lh_S_circular_insula_inf_LGI** | Equal variances not assumed |  |  | 0.071 | 25.509 | 0.944 | 0.00831 | 0.11752 | -0.23348 | 0.25010 |
| **lh_S_circular_insula_sup_LGI** | Equal variances assumed | 0.398 | 0.530 | -0.355 | 68 | 0.724 | -0.04010 | 0.11303 | -0.26565 | 0.18546 |
| **lh_S_circular_insula_sup_LGI** | Equal variances not assumed |  |  | -0.333 | 24.614 | 0.742 | -0.04010 | 0.12031 | -0.28807 | 0.20788 |
| **lh_S_collat_transv_ant_LGI** | Equal variances assumed | 0.345 | 0.559 | -0.957 | 68 | 0.342 | -0.03316 | 0.03466 | -0.10233 | 0.03601 |
| **lh_S_collat_transv_ant_LGI** | Equal variances not assumed |  |  | -0.929 | 25.854 | 0.361 | -0.03316 | 0.03568 | -0.10651 | 0.04019 |
| **lh_S_collat_transv_post_LGI** | Equal variances assumed | 4.194 | 0.044 | -0.733 | 68 | 0.466 | -0.02596 | 0.03544 | -0.09669 | 0.04476 |
| **lh_S_collat_transv_post_LGI** | Equal variances not assumed |  |  | -0.907 | 41.992 | 0.370 | -0.02596 | 0.02862 | -0.08372 | 0.03180 |
| **lh_S_front_inf_LGI** | Equal variances assumed | 1.588 | 0.212 | 0.075 | 68 | 0.941 | 0.00531 | 0.07090 | -0.13617 | 0.14678 |
| **lh_S_front_inf_LGI** | Equal variances not assumed |  |  | 0.084 | 33.440 | 0.934 | 0.00531 | 0.06323 | -0.12328 | 0.13389 |
| **lh_S_front_middle_LGI** | Equal variances assumed | 0.291 | 0.591 | -0.488 | 68 | 0.627 | -0.02152 | 0.04412 | -0.10956 | 0.06651 |
| **lh_S_front_middle_LGI** | Equal variances not assumed |  |  | -0.475 | 25.949 | 0.639 | -0.02152 | 0.04530 | -0.11465 | 0.07160 |
| **lh_S_front_sup_LGI** | Equal variances assumed | 0.339 | 0.562 | -0.392 | 68 | 0.696 | -0.01908 | 0.04870 | -0.11625 | 0.07810 |
| **lh_S_front_sup_LGI** | Equal variances not assumed |  |  | -0.404 | 28.531 | 0.689 | -0.01908 | 0.04719 | -0.11565 | 0.07750 |
| **lh_S_interm_prim-Jensen_LGI** | Equal variances assumed | 0.565 | 0.455 | -1.306 | 68 | 0.196 | -0.07155 | 0.05479 | -0.18089 | 0.03779 |
| **lh_S_interm_prim-Jensen_LGI** | Equal variances not assumed |  |  | -1.465 | 33.488 | 0.152 | -0.07155 | 0.04884 | -0.17085 | 0.02775 |
| **lh_S_intrapariet_and_P_trans_LGI** | Equal variances assumed | 0.116 | 0.735 | -1.101 | 68 | 0.275 | -0.05386 | 0.04894 | -0.15151 | 0.04379 |
| **lh_S_intrapariet_and_P_trans_LGI** | Equal variances not assumed |  |  | -1.163 | 29.776 | 0.254 | -0.05386 | 0.04630 | -0.14844 | 0.04073 |
| **lh_S_oc_middle_and_Lunatus_LGI** | Equal variances assumed | 1.117 | 0.294 | -0.009 | 68 | 0.993 | -0.00046 | 0.05141 | -0.10304 | 0.10212 |
| **lh_S_oc_middle_and_Lunatus_LGI** | Equal variances not assumed |  |  | -0.010 | 34.393 | 0.992 | -0.00046 | 0.04524 | -0.09236 | 0.09144 |
| **lh_S_oc_sup_and_transversal_LGI** | Equal variances assumed | 0.816 | 0.369 | -0.257 | 68 | 0.798 | -0.01235 | 0.04807 | -0.10826 | 0.08357 |
| **lh_S_oc_sup_and_transversal_LGI** | Equal variances not assumed |  |  | -0.241 | 24.563 | 0.812 | -0.01235 | 0.05123 | -0.11796 | 0.09327 |
| **lh_S_occipital_ant_LGI** | Equal variances assumed | 0.003 | 0.960 | -0.663 | 68 | 0.510 | -0.04367 | 0.06589 | -0.17515 | 0.08780 |
| **lh_S_occipital_ant_LGI** | Equal variances not assumed |  |  | -0.668 | 27.399 | 0.510 | -0.04367 | 0.06538 | -0.17773 | 0.09038 |
| **lh_S_oc-temp_lat_LGI** | Equal variances assumed | 0.384 | 0.537 | -0.413 | 68 | 0.681 | -0.01696 | 0.04103 | -0.09883 | 0.06490 |
| **lh_S_oc-temp_lat_LGI** | Equal variances not assumed |  |  | -0.373 | 23.306 | 0.713 | -0.01696 | 0.04547 | -0.11096 | 0.07704 |
| **lh_S_oc-temp_med_and_Lingual_LGI** | Equal variances assumed | 1.498 | 0.225 | 1.027 | 68 | 0.308 | 0.03753 | 0.03656 | -0.03541 | 0.11048 |
| **lh_S_oc-temp_med_and_Lingual_LGI** | Equal variances not assumed |  |  | 1.184 | 35.494 | 0.244 | 0.03753 | 0.03171 | -0.02681 | 0.10188 |
| **lh_S_orbital_lateral_LGI** | Equal variances assumed | 0.503 | 0.481 | 0.070 | 68 | 0.945 | 0.00469 | 0.06719 | -0.12939 | 0.13876 |
| **lh_S_orbital_lateral_LGI** | Equal variances not assumed |  |  | 0.074 | 29.638 | 0.942 | 0.00469 | 0.06373 | -0.12554 | 0.13491 |
| **lh_S_orbital_med-olfact_LGI** | Equal variances assumed | 0.931 | 0.338 | 0.202 | 68 | 0.840 | 0.00626 | 0.03096 | -0.05553 | 0.06805 |
| **lh_S_orbital_med-olfact_LGI** | Equal variances not assumed |  |  | 0.228 | 33.957 | 0.821 | 0.00626 | 0.02742 | -0.04945 | 0.06198 |
| **lh_S_orbital-H_Shaped_LGI** | Equal variances assumed | 0.754 | 0.388 | -0.637 | 68 | 0.527 | -0.02939 | 0.04617 | -0.12151 | 0.06273 |
| **lh_S_orbital-H_Shaped_LGI** | Equal variances not assumed |  |  | -0.695 | 31.711 | 0.492 | -0.02939 | 0.04226 | -0.11551 | 0.05673 |
| **lh_S_parieto_occipital_LGI** | Equal variances assumed | 0.067 | 0.797 | -0.662 | 68 | 0.510 | -0.04238 | 0.06399 | -0.17006 | 0.08530 |
| **lh_S_parieto_occipital_LGI** | Equal variances not assumed |  |  | -0.675 | 27.933 | 0.505 | -0.04238 | 0.06277 | -0.17096 | 0.08621 |
| **lh_S_pericallosal_LGI** | Equal variances assumed | 0.003 | 0.954 | 0.706 | 68 | 0.483 | 0.02347 | 0.03324 | -0.04286 | 0.08980 |
| **lh_S_pericallosal_LGI** | Equal variances not assumed |  |  | 0.713 | 27.516 | 0.482 | 0.02347 | 0.03290 | -0.04398 | 0.09092 |
| **lh_S_postcentral_LGI** | Equal variances assumed | 0.316 | 0.576 | -1.299 | 68 | 0.198 | -0.06906 | 0.05315 | -0.17512 | 0.03701 |
| **lh_S_postcentral_LGI** | Equal variances not assumed |  |  | -1.506 | 35.919 | 0.141 | -0.06906 | 0.04586 | -0.16207 | 0.02396 |
| **lh_S_precentral-inf-part_LGI** | Equal variances assumed | 0.304 | 0.583 | -0.502 | 68 | 0.618 | -0.04563 | 0.09099 | -0.22720 | 0.13593 |
| **lh_S_precentral-inf-part_LGI** | Equal variances not assumed |  |  | -0.561 | 33.244 | 0.579 | -0.04563 | 0.08138 | -0.21116 | 0.11989 |
| **lh_S_precentral-sup-part_LGI** | Equal variances assumed | 0.732 | 0.395 | -2.376 | 68 | 0.020 | -0.13390 | 0.05635 | -0.24634 | -0.02146 |
| **lh_S_precentral-sup-part_LGI** | Equal variances not assumed |  |  | -2.356 | 26.677 | 0.026 | -0.13390 | 0.05684 | -0.25059 | -0.01721 |
| **lh_S_suborbital_LGI** | Equal variances assumed | 1.609 | 0.209 | -0.525 | 68 | 0.601 | -0.01625 | 0.03092 | -0.07794 | 0.04545 |
| **lh_S_suborbital_LGI** | Equal variances not assumed |  |  | -0.616 | 36.787 | 0.542 | -0.01625 | 0.02639 | -0.06973 | 0.03724 |
| **lh_S_subparietal_LGI** | Equal variances assumed | 0.672 | 0.415 | 0.048 | 68 | 0.962 | 0.00270 | 0.05668 | -0.11041 | 0.11581 |
| **lh_S_subparietal_LGI** | Equal variances not assumed |  |  | 0.043 | 23.159 | 0.966 | 0.00270 | 0.06314 | -0.12786 | 0.13327 |
| **lh_S_temporal_inf_LGI** | Equal variances assumed | 0.944 | 0.335 | -0.480 | 68 | 0.633 | -0.02480 | 0.05163 | -0.12783 | 0.07824 |
| **lh_S_temporal_inf_LGI** | Equal variances not assumed |  |  | -0.444 | 24.104 | 0.661 | -0.02480 | 0.05579 | -0.13992 | 0.09033 |
| **lh_S_temporal_sup_LGI** | Equal variances assumed | 0.003 | 0.960 | -0.321 | 68 | 0.750 | -0.02330 | 0.07267 | -0.16830 | 0.12171 |
| **lh_S_temporal_sup_LGI** | Equal variances not assumed |  |  | -0.339 | 29.741 | 0.737 | -0.02330 | 0.06880 | -0.16385 | 0.11726 |
| **lh_S_temporal_transverse_LGI** | Equal variances assumed | 0.251 | 0.618 | -0.190 | 68 | 0.850 | -0.02092 | 0.11009 | -0.24061 | 0.19876 |
| **lh_S_temporal_transverse_LGI** | Equal variances not assumed |  |  | -0.184 | 25.661 | 0.856 | -0.02092 | 0.11387 | -0.25514 | 0.21330 |
| **rh_G_and_S_frontomargin_LGI** | Equal variances assumed | 3.332 | 0.072 | -0.219 | 68 | 0.827 | -0.00801 | 0.03654 | -0.08092 | 0.06491 |
| **rh_G_and_S_frontomargin_LGI** | Equal variances not assumed |  |  | -0.292 | 50.584 | 0.772 | -0.00801 | 0.02743 | -0.06309 | 0.04707 |
| **rh_G_and_S_occipital_inf_LGI** | Equal variances assumed | 3.661 | 0.060 | 0.114 | 68 | 0.910 | 0.00555 | 0.04890 | -0.09203 | 0.10314 |
| **rh_G_and_S_occipital_inf_LGI** | Equal variances not assumed |  |  | 0.147 | 46.617 | 0.884 | 0.00555 | 0.03789 | -0.07069 | 0.08180 |
| **rh_G_and_S_paracentral_LGI** | Equal variances assumed | 0.790 | 0.377 | -0.913 | 68 | 0.365 | -0.02854 | 0.03127 | -0.09094 | 0.03386 |
| **rh_G_and_S_paracentral_LGI** | Equal variances not assumed |  |  | -1.067 | 36.599 | 0.293 | -0.02854 | 0.02676 | -0.08278 | 0.02569 |
| **rh_G_and_S_subcentral_LGI** | Equal variances assumed | 1.381 | 0.244 | -0.862 | 68 | 0.392 | -0.09361 | 0.10854 | -0.31020 | 0.12299 |
| **rh_G_and_S_subcentral_LGI** | Equal variances not assumed |  |  | -0.810 | 24.593 | 0.426 | -0.09361 | 0.11560 | -0.33188 | 0.14467 |
| **rh_G_and_S_transv_frontopol_LGI** | Equal variances assumed | 1.612 | 0.209 | -0.436 | 68 | 0.664 | -0.01598 | 0.03666 | -0.08914 | 0.05717 |
| **rh_G_and_S_transv_frontopol_LGI** | Equal variances not assumed |  |  | -0.539 | 41.710 | 0.593 | -0.01598 | 0.02968 | -0.07590 | 0.04393 |
| **rh_G_and_S_cingul-Ant_LGI** | Equal variances assumed | 0.121 | 0.729 | -0.208 | 68 | 0.836 | -0.00672 | 0.03228 | -0.07114 | 0.05770 |
| **rh_G_and_S_cingul-Ant_LGI** | Equal variances not assumed |  |  | -0.227 | 31.628 | 0.822 | -0.00672 | 0.02959 | -0.06702 | 0.05358 |
| **rh_G_and_S_cingul-Mid-Ant_LGI** | Equal variances assumed | 0.517 | 0.475 | 0.022 | 68 | 0.982 | 0.00060 | 0.02729 | -0.05386 | 0.05506 |
| **rh_G_and_S_cingul-Mid-Ant_LGI** | Equal variances not assumed |  |  | 0.020 | 23.905 | 0.984 | 0.00060 | 0.02967 | -0.06066 | 0.06186 |
| **rh_G_and_S_cingul-Mid-Post_LGI** | Equal variances assumed | 0.361 | 0.550 | 0.169 | 68 | 0.866 | 0.00480 | 0.02835 | -0.05177 | 0.06137 |
| **rh_G_and_S_cingul-Mid-Post_LGI** | Equal variances not assumed |  |  | 0.188 | 32.653 | 0.852 | 0.00480 | 0.02558 | -0.04726 | 0.05686 |
| **rh_G_cingul-Post-dorsal_LGI** | Equal variances assumed | 1.407 | 0.240 | 0.961 | 68 | 0.340 | 0.05353 | 0.05571 | -0.05763 | 0.16469 |
| **rh_G_cingul-Post-dorsal_LGI** | Equal variances not assumed |  |  | 1.184 | 41.468 | 0.243 | 0.05353 | 0.04521 | -0.03775 | 0.14480 |
| **rh_G_cingul-Post-ventral_LGI** | Equal variances assumed | 0.871 | 0.354 | 0.666 | 68 | 0.507 | 0.04065 | 0.06100 | -0.08107 | 0.16237 |
| **rh_G_cingul-Post-ventral_LGI** | Equal variances not assumed |  |  | 0.765 | 35.218 | 0.449 | 0.04065 | 0.05310 | -0.06713 | 0.14843 |
| **rh_G_cuneus_LGI** | Equal variances assumed | 0.834 | 0.364 | -0.066 | 68 | 0.948 | -0.00407 | 0.06174 | -0.12728 | 0.11913 |
| **rh_G_cuneus_LGI** | Equal variances not assumed |  |  | -0.075 | 34.298 | 0.941 | -0.00407 | 0.05441 | -0.11462 | 0.10647 |
| **rh_G_front_inf-Opercular_LGI** | Equal variances assumed | 0.720 | 0.399 | 0.098 | 68 | 0.922 | 0.01205 | 0.12319 | -0.23378 | 0.25788 |
| **rh_G_front_inf-Opercular_LGI** | Equal variances not assumed |  |  | 0.093 | 25.071 | 0.927 | 0.01205 | 0.12944 | -0.25451 | 0.27860 |
| **rh_G_front_inf-Orbital_LGI** | Equal variances assumed | 0.202 | 0.655 | -0.698 | 68 | 0.488 | -0.08278 | 0.11861 | -0.31946 | 0.15390 |
| **rh_G_front_inf-Orbital_LGI** | Equal variances not assumed |  |  | -0.661 | 24.877 | 0.515 | -0.08278 | 0.12530 | -0.34091 | 0.17535 |
| **rh_G_front_inf-Triangul_LGI** | Equal variances assumed | 0.292 | 0.591 | 0.627 | 68 | 0.533 | 0.06519 | 0.10391 | -0.14216 | 0.27253 |
| **rh_G_front_inf-Triangul_LGI** | Equal variances not assumed |  |  | 0.614 | 26.114 | 0.545 | 0.06519 | 0.10625 | -0.15317 | 0.28354 |
| **rh_G_front_middle_LGI** | Equal variances assumed | 0.359 | 0.551 | -0.095 | 68 | 0.924 | -0.00508 | 0.05331 | -0.11145 | 0.10129 |
| **rh_G_front_middle_LGI** | Equal variances not assumed |  |  | -0.094 | 26.402 | 0.926 | -0.00508 | 0.05413 | -0.11626 | 0.10609 |
| **rh_G_front_sup_LGI** | Equal variances assumed | 0.402 | 0.528 | -0.512 | 68 | 0.611 | -0.01581 | 0.03091 | -0.07749 | 0.04586 |
| **rh_G_front_sup_LGI** | Equal variances not assumed |  |  | -0.489 | 25.262 | 0.629 | -0.01581 | 0.03231 | -0.08232 | 0.05070 |
| **rh_G_Ins_lg_and_S_cent_ins_LGI** | Equal variances assumed | 0.457 | 0.502 | -0.051 | 68 | 0.960 | -0.00664 | 0.13043 | -0.26691 | 0.25362 |
| **rh_G_Ins_lg_and_S_cent_ins_LGI** | Equal variances not assumed |  |  | -0.050 | 26.303 | 0.960 | -0.00664 | 0.13275 | -0.27937 | 0.26608 |
| **rh_G_insular_short_LGI** | Equal variances assumed | 0.799 | 0.375 | 0.041 | 68 | 0.968 | 0.00522 | 0.12805 | -0.25030 | 0.26075 |
| **rh_G_insular_short_LGI** | Equal variances not assumed |  |  | 0.039 | 25.529 | 0.969 | 0.00522 | 0.13291 | -0.26822 | 0.27866 |
| **rh_G_occipital_middle_LGI** | Equal variances assumed | 1.646 | 0.204 | -0.305 | 68 | 0.761 | -0.01616 | 0.05292 | -0.12175 | 0.08944 |
| **rh_G_occipital_middle_LGI** | Equal variances not assumed |  |  | -0.362 | 37.847 | 0.719 | -0.01616 | 0.04462 | -0.10650 | 0.07418 |
| **rh_G_occipital_sup_LGI** | Equal variances assumed | 0.001 | 0.981 | -1.252 | 68 | 0.215 | -0.06650 | 0.05311 | -0.17249 | 0.03949 |
| **rh_G_occipital_sup_LGI** | Equal variances not assumed |  |  | -1.165 | 24.281 | 0.255 | -0.06650 | 0.05709 | -0.18425 | 0.05125 |
| **rh_G_oc-temp_lat-fusifor_LGI** | Equal variances assumed | 0.066 | 0.798 | -0.026 | 68 | 0.979 | -0.00079 | 0.03015 | -0.06096 | 0.05938 |
| **rh_G_oc-temp_lat-fusifor_LGI** | Equal variances not assumed |  |  | -0.027 | 28.455 | 0.979 | -0.00079 | 0.02926 | -0.06069 | 0.05911 |
| **rh_G_oc-temp_med-Lingual_LGI** | Equal variances assumed | 3.727 | 0.058 | 0.258 | 68 | 0.797 | 0.01342 | 0.05201 | -0.09037 | 0.11720 |
| **rh_G_oc-temp_med-Lingual_LGI** | Equal variances not assumed |  |  | 0.340 | 49.238 | 0.735 | 0.01342 | 0.03945 | -0.06586 | 0.09269 |
| **rh_G_oc-temp_med-Parahip_LGI** | Equal variances assumed | 1.099 | 0.298 | 0.491 | 68 | 0.625 | 0.01818 | 0.03700 | -0.05565 | 0.09201 |
| **rh_G_oc-temp_med-Parahip_LGI** | Equal variances not assumed |  |  | 0.586 | 38.339 | 0.561 | 0.01818 | 0.03102 | -0.04461 | 0.08097 |
| **rh_G_orbital_LGI** | Equal variances assumed | 0.015 | 0.904 | -0.426 | 68 | 0.671 | -0.01823 | 0.04278 | -0.10360 | 0.06713 |
| **rh_G_orbital_LGI** | Equal variances not assumed |  |  | -0.440 | 28.519 | 0.663 | -0.01823 | 0.04146 | -0.10309 | 0.06663 |
| **rh_G_pariet_inf-Angular_LGI** | Equal variances assumed | 0.029 | 0.864 | -0.399 | 68 | 0.691 | -0.02358 | 0.05912 | -0.14155 | 0.09440 |
| **rh_G_pariet_inf-Angular_LGI** | Equal variances not assumed |  |  | -0.395 | 26.647 | 0.696 | -0.02358 | 0.05968 | -0.14611 | 0.09896 |
| **rh_G_pariet_inf-Supramar_LGI** | Equal variances assumed | 0.508 | 0.479 | -0.903 | 68 | 0.370 | -0.05810 | 0.06431 | -0.18644 | 0.07024 |
| **rh_G_pariet_inf-Supramar_LGI** | Equal variances not assumed |  |  | -0.866 | 25.350 | 0.394 | -0.05810 | 0.06707 | -0.19614 | 0.07993 |
| **rh_G_parietal_sup_LGI** | Equal variances assumed | 0.016 | 0.900 | -1.383 | 68 | 0.171 | -0.06276 | 0.04539 | -0.15334 | 0.02781 |
| **rh_G_parietal_sup_LGI** | Equal variances not assumed |  |  | -1.353 | 26.132 | 0.188 | -0.06276 | 0.04639 | -0.15810 | 0.03257 |
| **rh_G_postcentral_LGI** | Equal variances assumed | 0.001 | 0.970 | -1.473 | 68 | 0.145 | -0.08225 | 0.05583 | -0.19366 | 0.02916 |
| **rh_G_postcentral_LGI** | Equal variances not assumed |  |  | -1.614 | 31.890 | 0.116 | -0.08225 | 0.05097 | -0.18608 | 0.02158 |
| **rh_G_precentral_LGI** | Equal variances assumed | 0.837 | 0.363 | -0.830 | 68 | 0.409 | -0.04789 | 0.05770 | -0.16302 | 0.06724 |
| **rh_G_precentral_LGI** | Equal variances not assumed |  |  | -0.953 | 35.170 | 0.347 | -0.04789 | 0.05026 | -0.14990 | 0.05412 |
| **rh_G_precuneus_LGI** | Equal variances assumed | 0.075 | 0.785 | -0.068 | 68 | 0.946 | -0.00304 | 0.04438 | -0.09160 | 0.08552 |
| **rh_G_precuneus_LGI** | Equal variances not assumed |  |  | -0.073 | 30.047 | 0.943 | -0.00304 | 0.04179 | -0.08837 | 0.08230 |
| **rh_G_rectus_LGI** | Equal variances assumed | 2.030 | 0.159 | -0.617 | 68 | 0.539 | -0.01841 | 0.02983 | -0.07794 | 0.04112 |
| **rh_G_rectus_LGI** | Equal variances not assumed |  |  | -0.761 | 41.585 | 0.451 | -0.01841 | 0.02419 | -0.06723 | 0.03041 |
| **rh_G_subcallosal_LGI** | Equal variances assumed | 0.017 | 0.898 | -0.268 | 68 | 0.790 | -0.00855 | 0.03191 | -0.07222 | 0.05512 |
| **rh_G_subcallosal_LGI** | Equal variances not assumed |  |  | -0.275 | 28.292 | 0.785 | -0.00855 | 0.03107 | -0.07216 | 0.05506 |
| **rh_G_temp_sup-G_T_transv_LGI** | Equal variances assumed | 0.580 | 0.449 | -0.403 | 68 | 0.688 | -0.04700 | 0.11661 | -0.27969 | 0.18570 |
| **rh_G_temp_sup-G_T_transv_LGI** | Equal variances not assumed |  |  | -0.396 | 26.296 | 0.695 | -0.04700 | 0.11871 | -0.29087 | 0.19688 |
| **rh_G_temp_sup-Lateral_LGI** | Equal variances assumed | 0.757 | 0.387 | -0.319 | 68 | 0.751 | -0.02667 | 0.08371 | -0.19372 | 0.14038 |
| **rh_G_temp_sup-Lateral_LGI** | Equal variances not assumed |  |  | -0.316 | 26.663 | 0.755 | -0.02667 | 0.08447 | -0.20010 | 0.14676 |
| **rh_G_temp_sup-Plan_polar_LGI** | Equal variances assumed | 0.046 | 0.832 | -0.431 | 68 | 0.668 | -0.03956 | 0.09169 | -0.22251 | 0.14340 |
| **rh_G_temp_sup-Plan_polar_LGI** | Equal variances not assumed |  |  | -0.445 | 28.518 | 0.660 | -0.03956 | 0.08886 | -0.22143 | 0.14232 |
| **rh_G_temp_sup-Plan_tempo_LGI** | Equal variances assumed | 0.037 | 0.848 | -1.265 | 68 | 0.210 | -0.10492 | 0.08296 | -0.27047 | 0.06063 |
| **rh_G_temp_sup-Plan_tempo_LGI** | Equal variances not assumed |  |  | -1.250 | 26.539 | 0.222 | -0.10492 | 0.08396 | -0.27733 | 0.06750 |
| **rh_G_temporal_inf_LGI** | Equal variances assumed | 0.001 | 0.979 | -0.786 | 68 | 0.435 | -0.02812 | 0.03579 | -0.09953 | 0.04329 |
| **rh_G_temporal_inf_LGI** | Equal variances not assumed |  |  | -0.760 | 25.656 | 0.454 | -0.02812 | 0.03702 | -0.10427 | 0.04802 |
| **rh_G_temporal_middle_LGI** | Equal variances assumed | 0.477 | 0.492 | -0.545 | 68 | 0.587 | -0.03034 | 0.05563 | -0.14134 | 0.08067 |
| **rh_G_temporal_middle_LGI** | Equal variances not assumed |  |  | -0.517 | 24.930 | 0.610 | -0.03034 | 0.05868 | -0.15120 | 0.09053 |
| **rh_Lat_Fis-ant-Horizont_LGI** | Equal variances assumed | 0.024 | 0.877 | -0.095 | 68 | 0.924 | -0.01185 | 0.12450 | -0.26028 | 0.23658 |
| **rh_Lat_Fis-ant-Horizont_LGI** | Equal variances not assumed |  |  | -0.092 | 25.589 | 0.928 | -0.01185 | 0.12901 | -0.27725 | 0.25355 |
| **rh_Lat_Fis-ant-Vertical_LGI** | Equal variances assumed | 0.160 | 0.690 | -0.294 | 68 | 0.770 | -0.03985 | 0.13573 | -0.31070 | 0.23100 |
| **rh_Lat_Fis-ant-Vertical_LGI** | Equal variances not assumed |  |  | -0.293 | 26.907 | 0.772 | -0.03985 | 0.13619 | -0.31934 | 0.23963 |
| **rh_Lat_Fis-post_LGI** | Equal variances assumed | 0.213 | 0.646 | -0.680 | 68 | 0.499 | -0.06813 | 0.10020 | -0.26808 | 0.13181 |
| **rh_Lat_Fis-post_LGI** | Equal variances not assumed |  |  | -0.684 | 27.331 | 0.500 | -0.06813 | 0.09958 | -0.27234 | 0.13607 |
| **rh_Pole_occipital_LGI** | Equal variances assumed | 0.180 | 0.673 | -0.957 | 68 | 0.342 | -0.03018 | 0.03153 | -0.09310 | 0.03275 |
| **rh_Pole_occipital_LGI** | Equal variances not assumed |  |  | -0.964 | 27.387 | 0.343 | -0.03018 | 0.03130 | -0.09435 | 0.03400 |
| **rh_Pole_temporal_LGI** | Equal variances assumed | 0.192 | 0.663 | -0.047 | 68 | 0.963 | -0.00176 | 0.03744 | -0.07648 | 0.07295 |
| **rh_Pole_temporal_LGI** | Equal variances not assumed |  |  | -0.050 | 29.731 | 0.961 | -0.00176 | 0.03545 | -0.07420 | 0.07067 |
| **rh_S_calcarine_LGI** | Equal variances assumed | 2.876 | 0.094 | 0.449 | 68 | 0.655 | 0.03115 | 0.06935 | -0.10724 | 0.16953 |
| **rh_S_calcarine_LGI** | Equal variances not assumed |  |  | 0.565 | 43.618 | 0.575 | 0.03115 | 0.05515 | -0.08004 | 0.14233 |
| **rh_S_central_LGI** | Equal variances assumed | 0.052 | 0.820 | -1.793 | 68 | 0.077 | -0.10092 | 0.05629 | -0.21326 | 0.01141 |
| **rh_S_central_LGI** | Equal variances not assumed |  |  | -2.008 | 33.377 | 0.053 | -0.10092 | 0.05025 | -0.20312 | 0.00127 |
| **rh_S_cingul-Marginalis_LGI** | Equal variances assumed | 3.784 | 0.056 | 0.555 | 68 | 0.581 | 0.02206 | 0.03974 | -0.05724 | 0.10136 |
| **rh_S_cingul-Marginalis_LGI** | Equal variances not assumed |  |  | 0.688 | 42.013 | 0.495 | 0.02206 | 0.03208 | -0.04268 | 0.08681 |
| **rh_S_circular_insula_ant_LGI** | Equal variances assumed | 0.607 | 0.439 | -0.579 | 68 | 0.564 | -0.06552 | 0.11312 | -0.29124 | 0.16021 |
| **rh_S_circular_insula_ant_LGI** | Equal variances not assumed |  |  | -0.536 | 24.118 | 0.597 | -0.06552 | 0.12218 | -0.31762 | 0.18659 |
| **rh_S_circular_insula_inf_LGI** | Equal variances assumed | 0.176 | 0.676 | 0.057 | 68 | 0.954 | 0.00684 | 0.11900 | -0.23063 | 0.24431 |
| **rh_S_circular_insula_inf_LGI** | Equal variances not assumed |  |  | 0.057 | 27.061 | 0.955 | 0.00684 | 0.11898 | -0.23727 | 0.25095 |
| **rh_S_circular_insula_sup_LGI** | Equal variances assumed | 1.355 | 0.249 | -0.260 | 68 | 0.795 | -0.03258 | 0.12515 | -0.28231 | 0.21715 |
| **rh_S_circular_insula_sup_LGI** | Equal variances not assumed |  |  | -0.252 | 25.711 | 0.803 | -0.03258 | 0.12928 | -0.29847 | 0.23331 |
| **rh_S_collat_transv_ant_LGI** | Equal variances assumed | 1.519 | 0.222 | -0.843 | 68 | 0.402 | -0.02839 | 0.03366 | -0.09556 | 0.03878 |
| **rh_S_collat_transv_ant_LGI** | Equal variances not assumed |  |  | -0.943 | 33.267 | 0.352 | -0.02839 | 0.03010 | -0.08960 | 0.03283 |
| **rh_S_collat_transv_post_LGI** | Equal variances assumed | 0.536 | 0.467 | -0.609 | 68 | 0.545 | -0.01764 | 0.02898 | -0.07547 | 0.04019 |
| **rh_S_collat_transv_post_LGI** | Equal variances not assumed |  |  | -0.631 | 28.787 | 0.533 | -0.01764 | 0.02794 | -0.07480 | 0.03952 |
| **rh_S_front_inf_LGI** | Equal variances assumed | 0.244 | 0.623 | 0.424 | 68 | 0.673 | 0.03202 | 0.07547 | -0.11858 | 0.18263 |
| **rh_S_front_inf_LGI** | Equal variances not assumed |  |  | 0.420 | 26.621 | 0.678 | 0.03202 | 0.07623 | -0.12450 | 0.18855 |
| **rh_S_front_middle_LGI** | Equal variances assumed | 0.288 | 0.594 | 0.172 | 68 | 0.864 | 0.00935 | 0.05438 | -0.09916 | 0.11786 |
| **rh_S_front_middle_LGI** | Equal variances not assumed |  |  | 0.197 | 34.836 | 0.845 | 0.00935 | 0.04758 | -0.08725 | 0.10595 |
| **rh_S_front_sup_LGI** | Equal variances assumed | 0.007 | 0.932 | -0.535 | 68 | 0.595 | -0.02723 | 0.05094 | -0.12889 | 0.07443 |
| **rh_S_front_sup_LGI** | Equal variances not assumed |  |  | -0.538 | 27.332 | 0.595 | -0.02723 | 0.05063 | -0.13105 | 0.07659 |
| **rh_S_interm_prim-Jensen_LGI** | Equal variances assumed | 0.019 | 0.892 | -0.916 | 68 | 0.363 | -0.05888 | 0.06431 | -0.18721 | 0.06945 |
| **rh_S_interm_prim-Jensen_LGI** | Equal variances not assumed |  |  | -0.921 | 27.327 | 0.365 | -0.05888 | 0.06392 | -0.18995 | 0.07219 |
| **rh_S_intrapariet_and_P_trans_LGI** | Equal variances assumed | 0.062 | 0.805 | -0.723 | 68 | 0.472 | -0.04265 | 0.05901 | -0.16040 | 0.07510 |
| **rh_S_intrapariet_and_P_trans_LGI** | Equal variances not assumed |  |  | -0.690 | 25.208 | 0.496 | -0.04265 | 0.06177 | -0.16982 | 0.08452 |
| **rh_S_oc_middle_and_Lunatus_LGI** | Equal variances assumed | 0.546 | 0.462 | -0.552 | 68 | 0.583 | -0.03056 | 0.05539 | -0.14108 | 0.07997 |
| **rh_S_oc_middle_and_Lunatus_LGI** | Equal variances not assumed |  |  | -0.616 | 33.151 | 0.542 | -0.03056 | 0.04961 | -0.13146 | 0.07035 |
| **rh_S_oc_sup_and_transversal_LGI** | Equal variances assumed | 0.095 | 0.759 | -1.123 | 68 | 0.265 | -0.06380 | 0.05680 | -0.17714 | 0.04954 |
| **rh_S_oc_sup_and_transversal_LGI** | Equal variances not assumed |  |  | -1.123 | 27.045 | 0.271 | -0.06380 | 0.05681 | -0.18036 | 0.05276 |
| **rh_S_occipital_ant_LGI** | Equal variances assumed | 0.832 | 0.365 | 0.014 | 68 | 0.989 | 0.00093 | 0.06566 | -0.13009 | 0.13195 |
| **rh_S_occipital_ant_LGI** | Equal variances not assumed |  |  | 0.016 | 33.955 | 0.987 | 0.00093 | 0.05814 | -0.11722 | 0.11908 |
| **rh_S_oc-temp_lat_LGI** | Equal variances assumed | 0.222 | 0.639 | -1.002 | 68 | 0.320 | -0.03947 | 0.03940 | -0.11810 | 0.03916 |
| **rh_S_oc-temp_lat_LGI** | Equal variances not assumed |  |  | -1.033 | 28.497 | 0.310 | -0.03947 | 0.03821 | -0.11767 | 0.03873 |
| **rh_S_oc-temp_med_and_Lingual_LGI** | Equal variances assumed | 0.477 | 0.492 | 0.579 | 68 | 0.564 | 0.02153 | 0.03715 | -0.05261 | 0.09566 |
| **rh_S_oc-temp_med_and_Lingual_LGI** | Equal variances not assumed |  |  | 0.645 | 32.988 | 0.523 | 0.02153 | 0.03335 | -0.04633 | 0.08938 |
| **rh_S_orbital_lateral_LGI** | Equal variances assumed | 1.581 | 0.213 | -0.016 | 68 | 0.987 | -0.00114 | 0.07233 | -0.14547 | 0.14319 |
| **rh_S_orbital_lateral_LGI** | Equal variances not assumed |  |  | -0.019 | 38.417 | 0.985 | -0.00114 | 0.06060 | -0.12377 | 0.12149 |
| **rh_S_orbital_med-olfact_LGI** | Equal variances assumed | 0.791 | 0.377 | -0.287 | 68 | 0.775 | -0.00825 | 0.02870 | -0.06552 | 0.04903 |
| **rh_S_orbital_med-olfact_LGI** | Equal variances not assumed |  |  | -0.324 | 33.761 | 0.748 | -0.00825 | 0.02548 | -0.06005 | 0.04355 |
| **rh_S_orbital-H_Shaped_LGI** | Equal variances assumed | 0.223 | 0.638 | -0.783 | 68 | 0.436 | -0.03259 | 0.04160 | -0.11559 | 0.05042 |
| **rh_S_orbital-H_Shaped_LGI** | Equal variances not assumed |  |  | -0.826 | 29.621 | 0.416 | -0.03259 | 0.03947 | -0.11324 | 0.04806 |
| **rh_S_parieto_occipital_LGI** | Equal variances assumed | 2.519 | 0.117 | -0.061 | 68 | 0.952 | -0.00404 | 0.06634 | -0.13642 | 0.12834 |
| **rh_S_parieto_occipital_LGI** | Equal variances not assumed |  |  | -0.076 | 43.133 | 0.940 | -0.00404 | 0.05299 | -0.11090 | 0.10282 |
| **rh_S_pericallosal_LGI** | Equal variances assumed | 0.009 | 0.925 | 0.968 | 68 | 0.336 | 0.02972 | 0.03069 | -0.03152 | 0.09097 |
| **rh_S_pericallosal_LGI** | Equal variances not assumed |  |  | 1.035 | 30.389 | 0.309 | 0.02972 | 0.02872 | -0.02891 | 0.08836 |
| **rh_S_postcentral_LGI** | Equal variances assumed | 0.000 | 0.985 | -1.576 | 68 | 0.120 | -0.09213 | 0.05845 | -0.20876 | 0.02451 |
| **rh_S_postcentral_LGI** | Equal variances not assumed |  |  | -1.647 | 29.172 | 0.110 | -0.09213 | 0.05593 | -0.20649 | 0.02223 |
| **rh_S_precentral-inf-part_LGI** | Equal variances assumed | 0.155 | 0.695 | -0.169 | 68 | 0.866 | -0.01452 | 0.08589 | -0.18592 | 0.15688 |
| **rh_S_precentral-inf-part_LGI** | Equal variances not assumed |  |  | -0.176 | 28.861 | 0.862 | -0.01452 | 0.08269 | -0.18367 | 0.15462 |
| **rh_S_precentral-sup-part_LGI** | Equal variances assumed | 1.534 | 0.220 | -0.108 | 68 | 0.914 | -0.00635 | 0.05873 | -0.12354 | 0.11083 |
| **rh_S_precentral-sup-part_LGI** | Equal variances not assumed |  |  | -0.123 | 34.294 | 0.903 | -0.00635 | 0.05175 | -0.11150 | 0.09879 |
| **rh_S_suborbital_LGI** | Equal variances assumed | 3.107 | 0.082 | -0.405 | 68 | 0.687 | -0.01219 | 0.03013 | -0.07232 | 0.04794 |
| **rh_S_suborbital_LGI** | Equal variances not assumed |  |  | -0.520 | 46.127 | 0.605 | -0.01219 | 0.02344 | -0.05938 | 0.03499 |
| **rh_S_subparietal_LGI** | Equal variances assumed | 1.481 | 0.228 | 0.884 | 68 | 0.380 | 0.05881 | 0.06654 | -0.07396 | 0.19159 |
| **rh_S_subparietal_LGI** | Equal variances not assumed |  |  | 1.093 | 41.789 | 0.281 | 0.05881 | 0.05383 | -0.04984 | 0.16747 |
| **rh_S_temporal_inf_LGI** | Equal variances assumed | 0.016 | 0.898 | 0.118 | 68 | 0.907 | 0.00523 | 0.04448 | -0.08353 | 0.09400 |
| **rh_S_temporal_inf_LGI** | Equal variances not assumed |  |  | 0.109 | 24.274 | 0.914 | 0.00523 | 0.04782 | -0.09340 | 0.10387 |
| **rh_S_temporal_sup_LGI** | Equal variances assumed | 0.065 | 0.799 | -0.389 | 68 | 0.699 | -0.02603 | 0.06691 | -0.15955 | 0.10750 |
| **rh_S_temporal_sup_LGI** | Equal variances not assumed |  |  | -0.417 | 30.633 | 0.679 | -0.02603 | 0.06236 | -0.15327 | 0.10122 |
| **rh_S_temporal_transverse_LGI** | Equal variances assumed | 0.019 | 0.890 | -0.688 | 68 | 0.494 | -0.08071 | 0.11733 | -0.31484 | 0.15341 |
| **rh_S_temporal_transverse_LGI** | Equal variances not assumed |  |  | -0.703 | 28.036 | 0.488 | -0.08071 | 0.11484 | -0.31595 | 0.15452 |

**Supplementary Figure 1.** Scatter plot depicting an unadjusted relationship between the small-worldness index (σ) and the positive symptom factor score. Patients with TR and non-TR are shown as grey and black dots respectively.

**Supplementary Figure 2.** Scatter plot depicts an association, adjusted for age and sex, and the positive symptom dimension, between the residuals of small-worldness index (σ) and positive symptom dimension. Patients with TR and non-TR are shown as grey and black dots respectively.


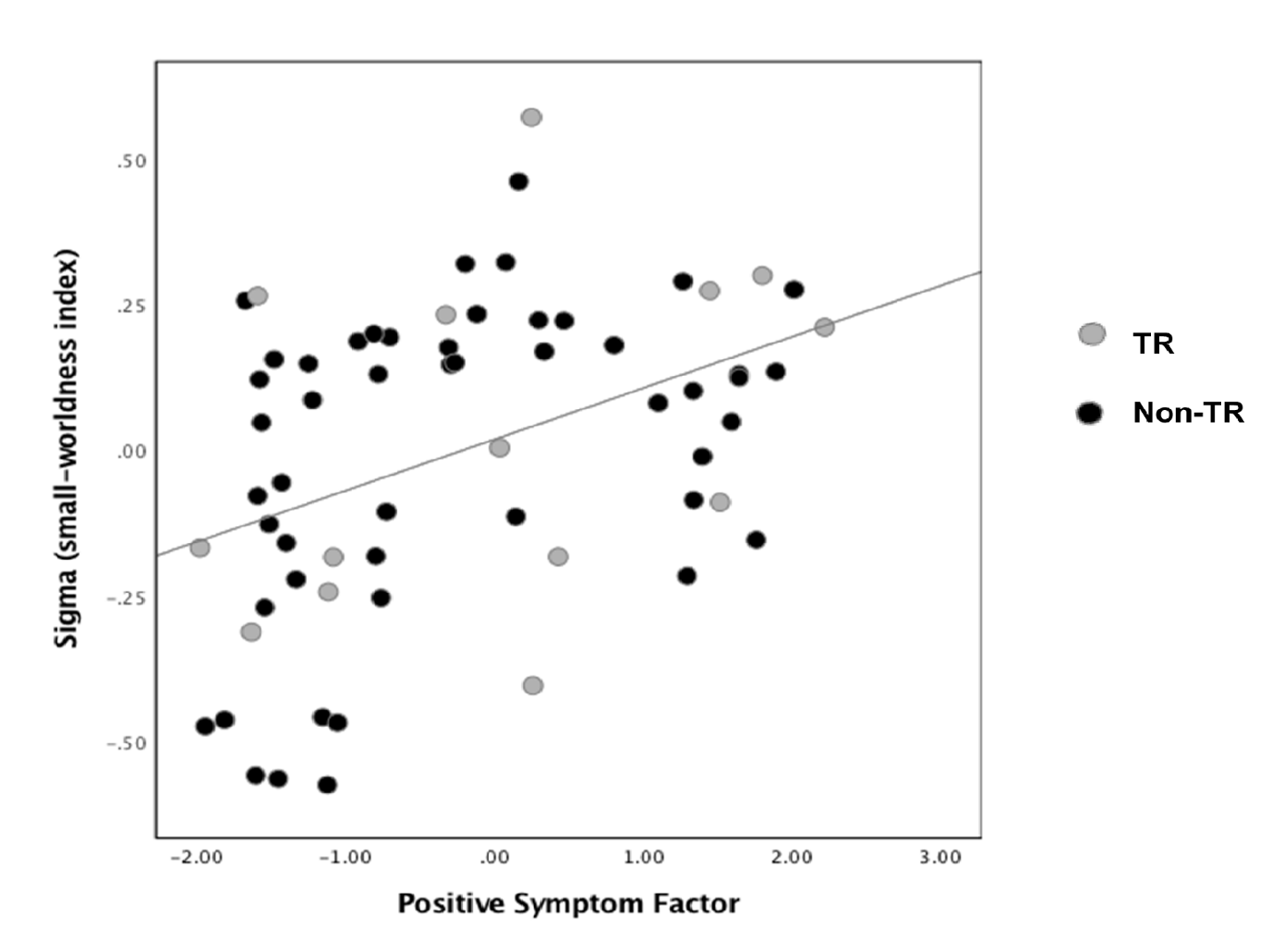

Supplement: sbab035_suppl_Supplementary_Materials [file sbab035_suppl_supplementary_materials.docx]
